# The Participatory Implications of Racialized Policy Feedback

**DOI:** 10.1017/s153759272100311x

**Published:** 2021-12-14

**Authors:** Sergio Garcia-Rios, Nazita Lajevardi, Kassra A. R. Oskooii, Hannah L. Walker

**Affiliations:** *assistant professor of government at Cornell University. His work has been featured in numerous outlets, including* Political Behavior *and* Sociological Methods and Research; *assistant professor of political science at Michigan State University and the author of* Outsiders at Home: The Politics of American Islamophobia. *Her work has been featured in the* Journal of Politics*, the* American Political Science Review*, and the* British Journal of Political Science*, among other outlets.*; *associate professor of political science and international relations at the University of Delaware. He has written more than 15 articles in esteemed outlets such as the* British Journal of Political Science *and* Public Opinion Quarterly; *assistant professor of government at the University of Texas at Austin and the author of the award-winning book,* Mobilized by Injustice: Criminal Justice Contact, Political Participation and Race. *Her research has been published in numerous journals, including the* Journal of Politics *and* Perspectives on Politics

## Abstract

How do involuntary interactions with authoritarian institutions shape political engagement? The policy feedback literature suggests that interactions with authoritarian policies undercut political participation. However, research in racial and ethnic politics offers reason to believe that these experiences may increase citizens’ engagement. Drawing on group attachment and discrimination research, we argue that mobilization is contingent on individuals’ political psychological state. Relative to their counterparts, individuals with a politicized group identity will display higher odds of political engagement when exposed to authoritarian institutions. To evaluate our theory, we draw on the 2016 Collaborative Multiracial Post-Election Study to examine the experiences of Blacks, Latinos, and Asian Americans. For all subgroups and different types of institutions, we find that, for those with a politicized group identity, institutional contact is associated with higher odds of participation. Our research modifies the classic policy feedback framework, which neglects group-based narratives in the calculus of collective action.

In 2017, more than seven million households relied on some form of subsidized housing (Kingsley 2017). The rate of children involved in the child welfare system was 6 per 1,000 children in the population, and Black children were overrepresented (Databank 2019). In 2018, more than three million Americans received cash transfers through national or state programs (Office of Family Assistance 2018). At the end of 2016, four and a half million individuals were on probation or parole at the end of 2016, and nearly 30% of working-age adults had a criminal record (Friedman 2015; Kaeble 2018).

Thus, contact with public institutions is pervasive, particularly with those institutions that scholars characterize as authoritarian or paternal (Soss et al. 2011). Public institutions impart civic lessons to clients, serving “as sources of information and meaning, with implications for political learning” (Mettler and Soss 2004, 60). Interactions with public policies and actors send messages about the value of one’s civic voice and standing in the polity (Lerman and Weaver 2014; Maltby 2017; Mettler 2005; Rocha, Knoll, and Wrinkle 2015; Soss 1999). Authoritarian or paternal policies, whose reach is extensive, have extraordinary consequences for US democracy by the lessons they teach and the citizens they make (Justice and Meares 2014; Lerman and Weaver 2014; Meares 2016).

Yet how do involuntary interactions with authoritarian institutions shape political engagement? Are citizens similarly affected by these interactions, or do responses vary by race? Policy feedback scholars answer the first question by examining a policy’s impact on an individual’s material and attitudinal resources, such as political efficacy and trust in government (Campbell 2002; Lerman and Weaver 2014; Mettler 2002; Michener 2018; Soss 1999). Such scholarship places institutional structure at the center of questions of engagement, distinguishing between those that empower individuals, encourage collective action, and engender efficacy and, alternatively, those that disempower and impede the development of civic capacity. Democratic policies that provide goods and services without excessive conditions and encourage participation in policy structure and implementation enable engagement and confidence in one’s role in the political milieu (Mettler and Soss 2004; Soss and Jacobs 2009).^1^ In contrast, interactions with authoritarian or paternal policies—characterized by top-down implementation, behavioral monitoring, and sanctions—degrade civic trust and capacity (Ryo 2016) and undermine participation (Lerman and Weaver 2014; Maltby 2017; Soss 1999).

Yet, although feedback scholars “study processes that are shot through with racial implications,” they often do so “without enough theoretically grounded consideration of race” (Michener 2019, 444). Indeed, research on minority participation offers reasons to believe that experiences with authoritarian policies may increase—rather than decrease—engagement. This work finds that individuals mobilize in defense of their rights when they believe they have been treated unfairly on account of their race, ethnicity, or other group affiliation (Miller et al. 1981; Parker 2009; Shingles 1981). A politicized group identity that views the state as a source of persistent inequality helps cultivate positive self-efficacy that encourages involvement in defending one’s rights and enhancing the status of one’s group. Some findings within the feedback literature support this idea. Soss (2005) highlights the response of some of the AFDC recipients he interviewed: they leveraged their sympathetic status as mothers toward a collective consciousness, rejecting the stigma associated with welfare receipt. In her study of the feedback effects of Medicaid, Michener (2018) notes what she terms *particular resistance* among beneficiaries who act to defend threatened services. Both authors direct policy feedback scholars to further examine the conditions that foster an embrace of political agency. Soss (2005, 322) explicitly directs us toward a dialogue with political psychology, writing that “a true dialogue with political psychology … remains an unrealized, and, to my mind, crucial goal for research on target groups.” In subsequent work, Michener (2019) provides a framework for understanding how and when we should explicitly engage racial and ethnic politics research.

We take up these scholars’ calls, both to examine the conditions under which individuals contest authoritarian policies and to bring racial and ethnic politics research into conversation with policy feedback theory. We argue that interactions with authoritarian institutions can be mobilizing when individuals hold a politicized group identity to which they connect their experience. Our central claim, then, is that a politicized group identity is an important fault line in determining whether authoritarian policies assist or attenuate participation. Thus, our theory should hold across a variety of authoritarian institutions. Finally, we examine how these relationships vary across racial subgroups. Although group consciousness is most coherent for Black Americans (Dawson 1994; Watts Smith, Lopez Bunyasi, and Smith 2019) and Latinos (Sanchez 2006; Zepeda-Millán 2017), research increasingly demonstrates that a group-based identity also can have meaningful political consequences for Asian Americans (Le, Arora, and Stout 2020; Wong, Lien, and Conway 2005).^2^ Thus, although race structures the nature of interactions with authoritarian policies and influences the likelihood of holding a politicized group identity, individuals who hold such an identity will be mobilized regardless of race.

To assess this theory, we leverage the 2016 Collaborative Multiracial Post-Election Study (CMPS). To our knowledge, the CMPS is the only dataset that permits an assessment of the relationship between interactions with authoritarian policies and political behavior across a number of institutions and racial groups and that also includes measures of a politicized group identity. We focus our analyses on the experiences of the three largest minoritized groups in the United States: Black Americans, Latinos, and Asian Americans. Perhaps unsurprisingly, Black Americans are both more likely to have contact with authoritarian institutions and to hold a politicized group identity than are Latinos and Asian Americans. However, across all three groups, individuals who have contact with authoritarian institutions and possess a politicized group identity are significantly more likely to participate politically than those without a politicized group identity. Among this second group, authoritarian encounters are negatively associated with participation. These findings are consistent across a number of institutions, among all three racial groups, and are robust to a variety of model specifications.

Our research makes several contributions. Heeding Soss (2005) and Michener (2019), we engage research on the political psychology of race to address a classic question about political learning. As such, we develop an alternative set of expectations for behavioral outcomes than would follow from the policy feedback literature, thus modifying the framework. Emerging work has taken initial steps to theorize about the mobilizing capacity of criminal justice (Walker 2020), but existing theory and evidence provide reason to think criminal justice is exceptional (Schneider and Ingram 1993). We argue that the logic of mobilization should extend to a class of related authoritarian institutions. Finally, very little feedback research examines differences among racial subgroups, and the handful of studies that do limit institutional variation. Yet, minorities in the United States interact with myriad institutions that cumulatively contribute to the daily experience of racialization, resulting in a particular kind of politics.

Taken as a whole, policy feedback scholarship suggests little capacity among citizens to mobilize in response to authoritarian policies. This perspective obscures important instances of collective action among minorities in the United States to counter state oppression. Drawing on the wealth of knowledge around racial and ethnic politics, we centralize the agency of the marginalized and offer alternative expectations for scholars leveraging policy feedback theory. We conclude the article with a discussion of our findings and a plan for future research.

## Literature and Theory

Our study enters into conversation with the policy feedback literature, extending it in three key ways. First, Michener (2019) critiques this body of work for its too infrequent attention to race as a central axis around which policy turns and its dearth of linkages to the racial and ethnic politics literature. Racial and ethnics politics literature should guide our theorizing about the behavioral consequences of a policy when it distributes benefits/burdens in racially disproportionate ways (Michener 2019). We build on Bruch, Ferree, and Soss’s (2010) insight that feedback effects among citizens hinge on whether a policy is administered in ways that are either authoritarian or democratic. We are specifically interested in the behavioral impacts of a class of authoritarian policies whose racial causes and consequences betray their stated liberal underpinnings, rendering their authoritarian structure unacceptably undemocratic. However, following Soss (2005), we depart from Bruch and coauthors (2010) to graft what we know about the political psychology of race onto the classic feedback framework to develop an alternative set of behavioral expectations. This modification to the participatory calculation made by traditional feedback research is our primary contribution.

To help us understand how racially disparate policy impacts may yield the kind of collective disaffection about which racial and ethnic politics scholars have written, we draw on theories of legal estrangement (Bell 2016) and perceived injustice (Walker 2020) developed around US criminal justice. Given the liberal underpinnings of criminal justice policies, scholars have traditionally focused on the individual nature of citizen–state interactions—the individual officer and perpetrator—when thinking about how these interactions affect civic attitudes. Bell (2016) and Walker (2020) turn that logic on its head and theoretically recast criminal justice as a fundamentally collective project, both in how it is administered and in how it is experienced by citizens. We extend that theoretical insight to a set of policies that Bruch and coauthors (2010) might categorize as authoritarian and empirically demonstrate its collective implications. In so doing, we offer a deeper critique of structures that rely on an individualized logic for democratic legitimacy but instead serve to reproduce racial power (Katzenstein, Ibrahim, and Rubin 2010).

### Modifying the Policy Feedback Framework

Everyday interactions with government agents and institutions matter for individuals’ political attitudes and behaviors (Mettler and Soss 2004; Pierson 1993; Soss 1999). Experiencing the government firsthand through participation in public programs teaches lessons about one’s value as a citizen, one’s capacity to engage with public life, and the efficacy of doing so. Routine interactions with government—such as being stopped by the police, serving in the military, or attending public school—socialize individuals into politics. Government agencies are actively “defining membership; forging political cohesion and group divisions; building or undermining civic capacities; framing policy agendas, problems, and evaluations; and structuring, stimulating, and stalling political participation” (Mettler and Soss 2004, 55).

The programmatic distribution of public goods instrumentally shapes political preferences and behavior. Some public goods are distributed in ways that both enhance access to resources and foster the attitudes, skills, and civic orientation requisite to engagement. Other public goods are distributed in ways that undercut access to resources and foster what scholars have termed an “anti-politics,” or a broad withdrawal from political life (Weaver, Prowse, and Piston 2020, 609). Whether a policy or program is civically generative or degrading turns on the messaging conveyed by its administration, teaching individuals that they are “atomized individuals who must deal directly with government and bureaucracy to press their own claims or participants in a cooperative process joining with others to solve problems collectively for the common good” (Schneider and Ingram 1993, 341).

Temporary Assistance for Needy Families (TANF) offers an example of program design that individualizes policy experiences. Failure to fulfill work activities according to the terms of TANF can lead to reduced benefits, which in turn conveys to clients that loss of assistance is the consequence of one’s own personal choices. Policies that condition the receipt of resources or initiate sanctions based on individual behaviors silo citizens in their interactions with government, obscure shared experiences with public institutions, and effectively foster “atomized publics with little sense of what they have in common and at stake in politics and government” (Soss and Jacobs 2009, 110). By contrast, Head Start, an early childhood education program that encourages parental involvement and local governance, is an example of an administrative structure that casts participants as engaged collaborators (Bruch, Ferree, and Soss 2010). Programs like TANF that condition the receipt of goods on behavioral metrics are authoritarian or paternalistic, structured as they are to elicit compliance; the participatory and distributive nature of programs like Head Start are administratively democratic. Researchers argue that democratic policies enhance participation (e.g., Campbell 2002; Mettler 2002), whereas authoritarian policies degrade participation (e.g., Bruch and Soss 2018; Lerman and Weaver 2014; Soss 1999; White 2019).

However, the liberal logic girding authoritarian policies, by which individuals are excluded from goods and services or subject to sanctioning because of their noncompliant behavior, itself disrupts the too easy conclusion that they yield “a unilateral withdrawal from political activity” (Weaver et al. 2020, 609). This exclusive logic may be invoked to serve ascriptive ends, which Katzenstein and colleagues (2010) refer to as “hyphenate liberalism.” This illiberal turn is most glaringly apparent in the area of criminal justice: policies are both justified by citizens’ violation of the social contract and are so excessive and disparately applied that eroded belief in the legitimacy of law enforcement is widely understood as a problem for the institution (Justice and Meares 2014; Meares 2015; Meares, Tyler, and Gardener 2015). The racially disparate outcome of a program or policy is precisely the instance, moreover, when Michener (2019) admonishes feedback scholars both to address race and leverage racial and ethnic politics research to guide theorizing about how such policies might affect attitudes and behaviors. A large body of literature demonstrates that rejecting the belief that one’s negative experiences are a result of bad behavior, and instead locating those experiences in practices that target one based on group affiliation, is central to collective action (Dawson 1994; Junn 2006; Miller et al. 1981; Shingles 1981). Thus, the process of decoding liberal values as cover for illiberal policies creates the potential for heightened engagement rather than withdrawal.

Schneider and Ingram’s (1993) seminal typology of target populations, which motivates theories of political learning, portends this slippage in the feedback framework: they anticipate that policies targeted to deviants may compel disruptive action, such as striking and protesting, out of a sense of angry defiance directed at an abusive power structure. Scholars refer to disaffection developed from criminal legal experiences as “legal estrangement” (Bell 2016). Legal estrangement is by definition collective, deriving not just from one’s own experience but also from how carceral policies affect “your friends, your intimate partners, your parents, your children; to people of your race or social class; and to people in the neighborhood or city where you live”; as such, it “is not merely an individual feeling to which people of color tend to succumb more readily than White Americans do” but is rather “a collective institutional venture” (p. 2058). This recasting of individual experiences as collective ones changes the participatory equation under the feedback framework: when the message received from interactions with authoritarian institutions is that one’s experiences are a consequence of stigmatized ascriptive qualities, rather than personal choice, the problem is the policy itself and it is collective—and as such it holds the possibility for political mobilization. Indeed, referring to this psychological response to interactions with the criminal justice system as perceived injustice, Walker (2020) links it to heightened political engagement. Likewise, speculating about differences observed with respect to welfare (demobilizing) and policing (mobilizing), Lawless and Fox (2001) point to racial discrimination by police officers as a potential catalyst for action; Nuamah and Ogorzalek (2021) argue that geographically concentrated and racially disparate education policies can spark group consciousness, promoting mobilization; and Laniyonu (2019) finds increased voting in high stop-and-frisk communities in New York City when a candidate campaigned on reforming these excessive and racialized practices.

The recognition that policies disproportionately affect individuals on the basis of ascriptive characteristics is a necessary ingredient of mobilization, because “it presents violations of democratic norms of equality and fairness, and a potential threat to a group’s political, cultural, or economic status” (Oskooii 2020, 7). However, research on the participation of marginalized groups suggests that this kind of disaffection is not sufficient to promote political engagement, where in addition to estrangement, authoritarian practices may “incentivize strategic retreat from engagement with the state, broadly speaking” (Weaver et al. 2020, 606). Political action follows from experiences with ascriptively authoritarian policies when individuals hold a strong affinity with the group relevant in the given policy context, beyond basic group membership (Garcia-Rios, Pedraza, and Wilcox-Archuleta 2018; Pérez 2015; Valenzuela and Michelson 2016). A strong group identity boosts internal efficacy, indicates a group with whom to organize, and bolsters the belief in the importance of collective action (Dawson 1994; Miller et al. 1981; Sanchez 2006; Shingles 1981).

The relationship between the citizen and the state is therefore dynamic. Departing from the traditional feedback framework, which with few exceptions leads us to anticipate that interactions with authoritarian policies should degrade civic engagement, we instead expect that this relationship should be conditional on the strength of one’s group identity. Viewing one’s experiences with authoritarian policies as a consequence of group membership, in which individuals view themselves as having been targeted, treated unfairly, or otherwise disadvantaged by a policy, casts one’s experiences as collective rather than individual. Further perceiving one’s group membership in political terms can help individuals convert estrangement into action. This generates the following hypothesis:

H1: *For those who view themselves as targets of institutional discrimination or deprivation based on group membership and who view themselves as politically connected to that group, interactions with authoritarian institutions will be associated with greater odds of political participation.*

Viewing one’s group membership as a source of political agency is necessary to mobilization. Interpreting one’s experiences as a consequence of group membership without seeing that membership as a source of power leaves the individual siloed, politically vulnerable, and likely to withdraw, yielding the following:

H2: *For those who view themselves as targets of institutional discrimination or deprivation based on group membership but who do not view themselves as politically connected to that group, involuntary interactions with authoritarian institutions will be associated with relatively low levels of political participation.*

Legal estrangement or a sense of injustice indicates that one has decoded and rejected the individualized logic of exclusion embedded in authoritarian policies. A strong, politicized group identity may aid in this process, although it need not be preceded by nor catalyze such an identity. It may be that some people already have a strong group identity and that interactions with a given institution connected to that identity, like police for Black Americans, makes that identity salient. It may be that, for others, interacting with an institution educates them about their own subordinate racial status, promoting the development of a politicized identity. Both sets of processes are plausible but not mutually exclusive. Instead, it follows from the literature that when individuals are disaffected with authoritarian policies because of their ascriptive causes and consequences, and that disaffection is accompanied by a strong affinity for the relevant group given the policy context, political action is more likely.

### Generalizability of the Politics of Estrangement

Although Schneider and Ingram (1993) predict the mobilizing capacity of experiences with the criminal legal system, they see less potential for engagement for those who are constructed more sympathetically as dependents of the state. Indeed, existing literature, diverse and unruly as it is, offers reason to suspect that policies like policing beget a politics distinct from that generated by paternal welfare provision. Such policies might include means-tested programs like TANF and public housing; or it might include other government programs that are not explicitly authoritarian but that intervene in private life while providing services, such as child welfare or addiction services. Although policies targeted toward dependents may be authoritarian in structure, they deliver important material goods and may engender the feeling that one is pitiably needy and that government is justifiably or irrevocably hierarchical (Schneider and Ingram 1993). Interactions with government agents in welfare and public housing (Bruch, Ferree, and Soss 2010) may be less obviously ascriptively problematic than with policing (Lawless and Fox 2001). It may be that interactions with the criminal legal system are so consequential that they demand a response, regardless of group consciousness. As such, policies that are paternal but less severely disciplinarian perhaps wane in their capacity to politically mobilize.

Yet, there are compelling reasons to think that even recipients whom Schneider and Ingram (1993) characterize as dependent mobilize when their group identities are made salient by authoritarian policies. The logic of hyphenate liberalism extends to a class of policies for which individual choices are used as grounds for program exclusion and policy administration is raced and classed. Soss and coauthors (2011) note that the expansion of welfare under the War on Poverty originated in the civil rights movement and was advanced by the growing political power of Black Americans; they also claim that welfare masked contests over racial power. The backlash that followed culminated in welfare-to-work reforms and heightened behavioral monitoring of recipients; it also devolved administration to states and localities (Soss et al. 2011). Opponents to racial progress grafted Black pathologies alive in the public imagination to welfare, converting it to a vehicle for regulating the behavior of undeserving recipients. This authoritarian turn characterizes poverty governance, broadly speaking.

One’s interaction with an authoritarian policy need not be explicitly negative to invoke a politicized group identity. For example, in their examination of the impact of investigatory stops by police, Epp and coauthors (2014) find that Black Americans and Latinos felt violated by invasive stops regardless of officer disposition. The belief that a policy is facially problematic itself undermines the legitimacy of that policy. Epp and colleagues’ (2014) finding is curious in the face of both theories of procedural justice and the approach taken by some policy feedback scholars, given that both focus on *individual* interactions with state actors. The implicit expectation is that because negative interactions can yield particular attitudinal and behavioral outcomes, negative interactions are the impetus for such responses. Yet, Epp, Haider-Markel, and Maynard-Moody (2014), Bell (2016) and Walker (2020) each observe that criminal justice is a collective project and that the valence of any given interaction cannot overcome the belief that a policy is itself unequal, either by design or implementation. We argue both that the valence of one’s interaction with authoritarian institutions need not be explicitly negative to make salient a politicized group identity grounded in race and that the capacity to do so rests with a wide variety of institutions that might be characterized as authoritarian. From this we derive the following:

H3: *The positive/negative relationship between the absence/presence of a politicized group identity and political participation will not vary greatly by the type of authoritarian institution under study.*

We draw on theories of group consciousness to modify the behavioral expectations derived from existing policy feedback research. This raises the question: Does the relationship between interactions with authoritarian institutions and political behavior vary by race? Research among Black Americans is foundational to contemporary research on the politics of race (Junn and Masuoka 2008). Dawson (1994)’s theory of the *Black utility heuristic* asserts that African Americans who perceive that their fates are tied to their racial group leverage group-based interests to make political decisions. Although Black linked fate is not homogeneous, scholars agree that it is persistent and strong (Greer 2013; Smith 2014).

Scholars have assessed the applicability of linked fate to other racial groups. However, the experiences of other racial groups are rooted in histories and degrees of discrimination different from those of Black Americans, complicating widespread adherence to a group-based identity. The sociopolitical experiences of Latinos vary by national origin, citizenship, and histories with immigration, and these cleavages generate disparate political preferences (Lavariega Monforti and Sanchez 2010).^4^ Latino group consciousness is context dependent, is most salient with reference to issues that cross-cut the community, and is associated with increasing anti-immigrant sentiment, which indiscriminately targets all Latinos regardless of legal status (Zepeda-Millán and Wallace 2013). Even so, the percentage of Latinos with some sense of racial linked fate is close to that of Black Americans and has been linked to political mobilization (Sanchez 2006).

A common Asian American identity is similarly constructed without a unifying language, history, or culture (Le Espiritu 1992). Yet Asian Americans have been lumped together in the American context, a process that has not been without consequence. Given that immigration policies have historically favored the low-skilled and racialized tropes as a perpetual “other” pervade the sociopolitical imagination, scholars persuasively argue that a politicized group identity may motivate the political attitudes and behaviors for at least a subset of the population (Arora, Sadhwani, and Shah 2021; Junn and Masuoka 2008; Masuoka and Junn 2013). Moreover, social movements that bring issues of import to Asian Americans into mainstream politics help cultivate a shared panethnic identity among this group; indeed, Lien, Conway, and Wong’s (2004) analysis finds that the panethnic mobilization has been successful.

We therefore focus our attention on the three largest nonwhite groups in the United States: Black, Latino and Asian Americans. Although the source and strength of a group-based identity vary by race, research suggests that viewing oneself as politically connected to their group can mobilize irrespective of race. This generates the following implication:

H4: *The positive/negative relationship between the absence/presence of a politicized group identity and political participation will not vary by race.*

We leverage racial and ethnic politics research to rethink the implications derived from policy feedback theory around the impact of interactions with authoritarian institutions on political engagement. The feedback literature largely suggests that interactions with authoritarian policies degrade participation. Institutions convey the message that individuals’ experiences result from their personal choices, and this individualized logic renders the authoritarian structure of a given policy democratically palatable. However, feedback scholars also note that interactions with institutions are subject to interpretation and that individuals “in a single public program… may draw different lessons from their encounters with the same design elements” (Mettler and Soss 2004, 64). Estrangement developed from corroded civic trust exemplifies the interpretive aspect of political learning. Yet, when estrangement is rooted in the racially disparate nature of a policy and accompanied by a politicized group identity, it may mobilize. A politicized group identity can transform interactions with authoritarian policies from alienating to energizing, and citizens from “passive subjects acted on by authorities” (Weaver and Lerman 2010, 3) to active agents contending with the policies that govern their lives. Accounting for a politicized group identity creates the space to take seriously the political agency of the marginalized and generates an alternative set of participatory expectations. This alternative set of expectations should likewise apply across a class of related policies and among all racial subgroups. We now turn to testing this alternative set of expectations.

## Data and Methods

We draw on the 2016 Comparative Multiracial Post-Election Survey (CMPS) to test our theory. Surveys as a means of evaluating policy feedback effects are not ideal, because they often do not adequately sample individuals likely to have contact with authoritarian institutions. Furthermore, assessing the causal effects of a given policy with observational data requires a repeated cross-sectional or panel design, as some scholars have employed (Bruch and Soss 2018; Jacobs and Mettler 2018). However, no research that we know of assesses the feedback effects of a variety of institutions in a single study across multiple racial minority groups.^6^ No appropriately expansive dataset that would permit these kinds of comparisons existed before development of the CMPS.

Although the CMPS is not a panel dataset that would enable us to evaluate the causal impact of authoritarian policies on participation, it offers a unique opportunity to expand the scope of policy feedback research in ways not previously possible. The CMPS is the most fitting dataset for our inquiry because it contains (1) questions about levels of contact with a variety of political institutions; (2) known proxies for a politicized group identity; (3) sufficient over-samples to derive estimates with reasonable precision among racial subgroups; and (4) robust samples of unregistered and low-income individuals, who are more likely to interact with the types of institutions about which we are concerned.

The CMPS was conducted between December 3, 2016, and February 15, 2017, and includes 3,006 Asian Americans, 3,099 Black Americans, and 3,002 Latinos (total *N* = 9,107). Respondents could take the survey in one of the following languages: English, Spanish, Chinese (simplified or traditional), Korean, and Vietnamese. The sampling methodology used both list and density techniques; the mode of collection was online, and researchers employed an innovative random-recruit-to-web (RWW) approach that approximates random-digit dial sampling (Barreto et al. 2018). The result is the most comprehensive dataset in the study of racial and ethnic politics to date.^7^

The CMPS asked respondents whether they had engaged in a range of activities in the last 12 months: (1) discussed politics with loved ones, (2) worked on a political campaign, (3) donated money to a political organization, (4) sported campaign paraphernalia, (5) contacted a government official, (6) contacted a government office, (7) cooperated with others to solve a community problem, (8) attended a meeting to discuss issues facing the community, (9) protested, (10) signed a petition, or (11) boycotted a company or product for political reasons. We combined these items into an index ranging from 0–10 (α reliability coefficient of .803), with a mean of 1.71 activities and a standard deviation of 2.24.^8^

Readers may wonder why we did not evaluate each act independently and why we excluded voting from our analysis. We did not evaluate each act independently in the main body of the analysis because we do not have a theory about the kinds of activities that interactions with authoritarian institutions may engender; the literature suggests that individuals may participate in protests (e.g., Walker 2020), community-focused activities (e.g., Weaver et al. 2020), and, if the electoral context is right, elections (e.g., Laniyonu 2019). Likewise, evaluating the outcomes in this way yields a proliferation of models. We leverage this analysis as a robustness check, described later, and direct readers to the appropriate section of the online appendix. We elected not to evaluate voting because we include individuals in our analysis who may not have access to the vote, such as noncitizens and, in some states, people with felony convictions. Furthermore, the sample size of eligible voters by racial subgroups with a politicized group identity who reported at least one form of institutional contact is too small to draw any firm conclusions about electoral behavior.^9^

To capture contact with an assortment of related institutions we relied on the following battery: *How often have you had involuntary dealings with these government agencies or o*ffi*cials in your community?* The agencies inquired about included police or school resource officers, courts, probation or parole offices, bail offices, a halfway house or treatment facility, the local housing authority, the local jail or state prison, the child welfare system, and family court. Response options included “often” (coded as 3) to “sometimes,” “occasionally,” and “never” (coded as 0). This battery does not include all policies that might be classified as authoritarian: TANF and immigration are notable exclusions. However, the battery covers a wider range of related institutions than those strictly associated with criminal justice. Although individuals may enter treatment facilities as a result of criminal justice contact (Rosenberg, Groves, and Blankenship 2017), they also can enter such facilities for a variety of other reasons, such as a serious substance-related family problem, substance-related job problem, and concern by a medical professional (Weisner 1993). Experiences with the child welfare system and family court similarly are sometimes related to criminal justice (Roberts 2011),^10^ but many children are removed from their families for reasons of neglect and abuse that do not involve the criminal justice system (Chibnall et al. 2003). These dynamics are present in our data. For example, a correlation matrix of these variables indicates that while contact with police has a greater than .5 correlation with all other institutions, the highest correlation is with courts, at .67. No institution achieves greater than a .67 correlation with contact with policing. Involuntary interactions with child welfare are most strongly associated with family court at .75, and most weakly related to policing at .51.^11^ About 55% of individuals with frequent contact with police also have very frequent contact with bail, 57% with halfway housing, and 57% with probation. Yet, 34% of individuals who have contact with child welfare services never have contact with police, and 29% never have contact with housing authorities.^12^ In sum, contact with these institutions are related, and contact with one puts an individual at risk of contact with multiple institutions. However, the variation we find here suggests that the dynamics we examine in this article extend beyond the criminal legal system.

Finding consistency across institutions would offer support for the generalizability of our theory to other connected authoritarian policies not explicitly asked about. The distribution of contact with various institutions is reported in table 1. Respondents most frequently reported contact with police (35%). About 30% of respondents reported having had contact with the courts. Between 15 and 20% of respondents reported contact with all other institutions. Taking a look at contact more generally, we find that 34 percent of the total sample reported no contact with any institution. Black respondents were most likely to have had some contact: 58% reported contact with at least one institution. In comparison, 46% of Latinos and 32% of Asian Americans reported at least one type of contact.

We argue that the relationship between involuntary contact and participation is conditional on holding a politicized group identity, which has at least two key components: a psychological feeling of shared circumstances or commonality with one’s group members, and the perception that membership confers unique disadvantages, such as being targets of discrimination. For the first component of this concept, we rely on the following racial linked fate question that is standard in the literature: *Do you think what happens generally to [respondent racial group] people in this country will have something to do with what happens in your life?* Respondents who selected the “yes” response option were coded as 1, and those who stated “no” were assigned the value 0. Among the total sample, 62% of respondents reported that what happens to people in their racial group will have something to do with what happens in their lives. Among racial subgroups, 67% of Black Americans, 58% of Latinos, and 61% of Asian Americans reported a sense of linked fate or connection with their racial group.

To measure the second component—the perception of discrimination based on one’s group affiliation—we rely on a standard discrimination question used in prior studies that asks about group identity and discrimination (Sanchez 2006; Schildkraut 2005). Respondents were asked: *Have you ever been treated unfairly or personally experienced discrimination because of your race, ethnicity, being an immigrant, religious heritage or having an accent?*^13^ Fifty-three percent of the sample, including 64% of Black Americans, 49% of Latinos, and 47% of Asians, indicated having previously experienced discrimination.

The theoretical model suggests that individuals who have had involuntary contact with authoritarian institutions *and* posses a politicized group identity will display higher odds of political engagement compared to their counterparts who lack such an identity. We therefore evaluated the data by examining the moderating effect of perceived discrimination and involuntary contact on political participation among subsamples of those with and without linked fate. Because the outcome of interest is a count of the number of political activities in which one reports engaging, which ranges from 0 to 10, we evaluate these relationships using ordinary least squares (OLS) regression.^14^ All of our models account for standard control variables included in political participation studies: political interest, political efficacy (internal), worship attendance, identification with a political party, gender, age, education and income. Information on the distribution of all variables included in the analysis is located in online table A1, along with details on question wording and coding schemes.

**Figure 1:**
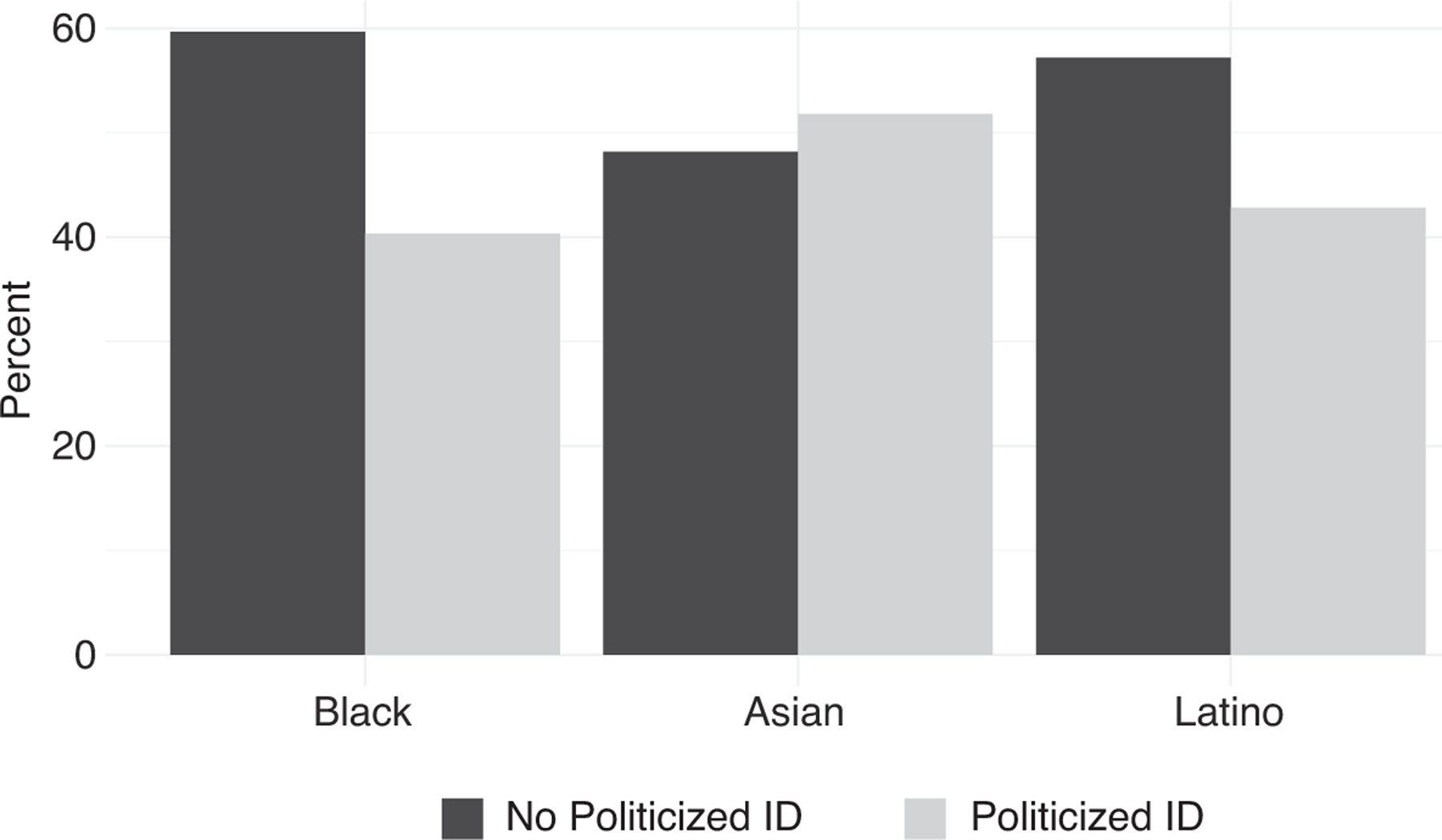
Presence and Absence of a Politicized Group Identity among Racial Subgroups Who Reported Any Involuntary Institutional Contact

Before outlining the findings, in figure 1 we report the proportion of respondents who reported any involuntary contact with authoritarian institutions by the absence and presence of a politicized group identity among each racial group. This comparison shows that, across all of the racial subgroups, a sizable percentage of respondents fall into both the politicized and nonpoliticized identity group categories, which enables us to draw reliable inferences about the interaction between involuntary contact and group identity on political participation. The descriptive statistics also demonstrate some variation among the three groups. Among Latinos and Asian Americans, a majority of respondents who had any contact do not possess a politicized group identity. However, a slight majority of Black Americans who reported any contact hold a politicized group identity. This difference is not surprising given that a higher proportion of Black American respondents reported a sense of linked fate and experiences with personal discrimination compared to their Latino and Asian American counterparts.

## Analysis and Results

**Figure 2:**
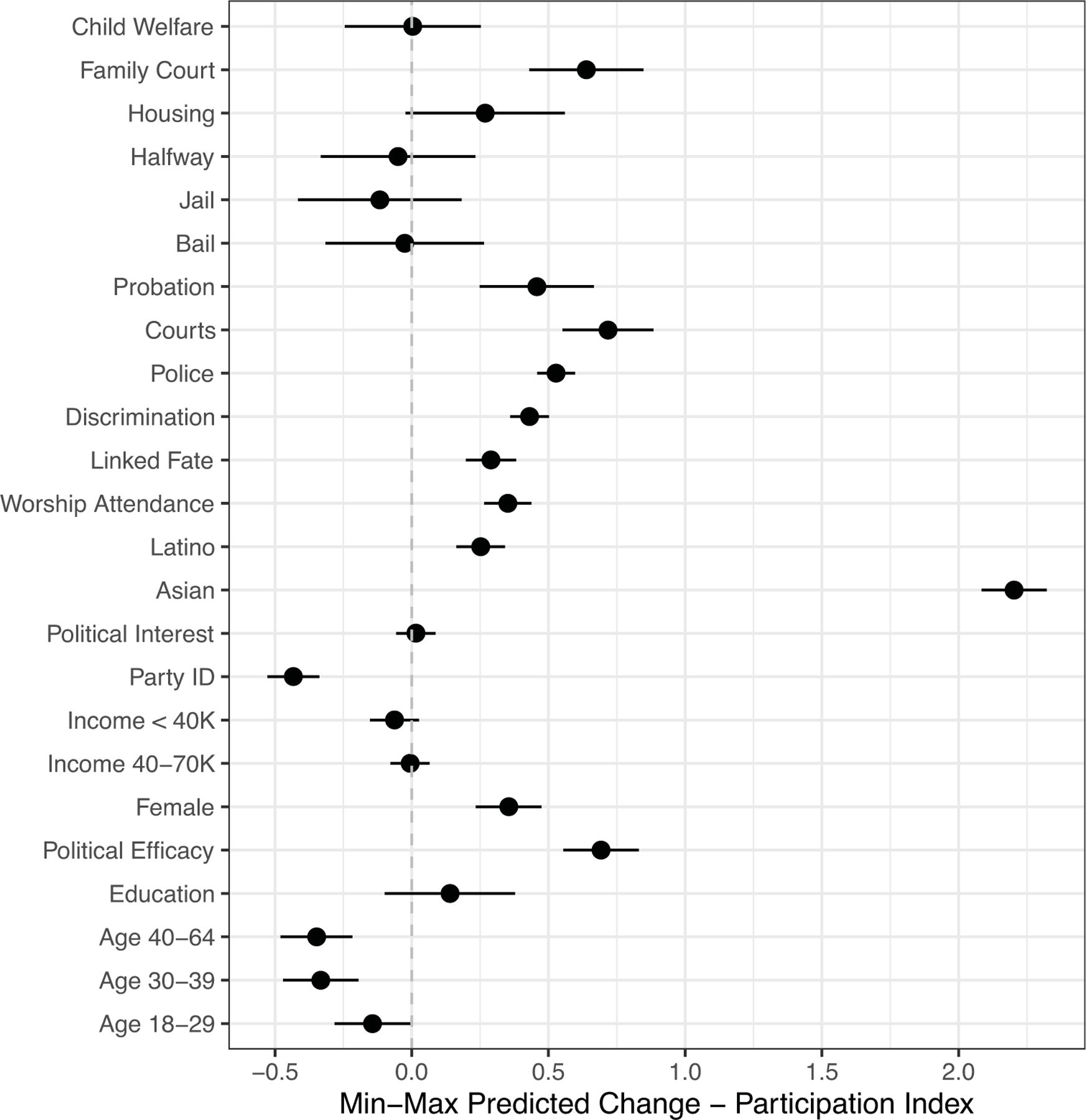
Involuntary Contact with Authoritarian Institutions and Political Participation *Note*: Simulated changes in predicted acts of political participation with 90% confidence bands correspond to regression results reported in online table A2.

We first examine the relationship between contact with various institutions and participation. Figure 2 displays the changes in predicted values of participation with 90% confidence bands for all variables included in the model. Coefficients and standard errors are displayed in online table A2. The findings suggest that several types of institutional contacts—including contact with public housing authorities, the courts, and the police—are positively associated with participation. Respondents who reported having often interacted with the police, for instance, were engaged in approximately one more political activity than those who did not report any contact with the police. To put things in perspective, education has a similar substantive impact on engagement. The results also demonstrate that both linked fate and perceived discrimination are positively associated with political participation.

Yet, even though contact with some institutions is positively associated with participation, the overall patterns are decidedly mixed. The positive association between contact with the police or the courts and participation does not extend to other criminal justice institutions. We also do not observe an association between contact with welfare or family court and participation. If we were to conclude our inquiry here, we would be left with perplexing conclusions regarding the relationship between institutional contact and political behavior. For example, we would be hard-pressed to explain why contact with probation and bail is not associated with participation, but contact with the housing authorities and the police is positively associated with engagement.

**Figure 3:**
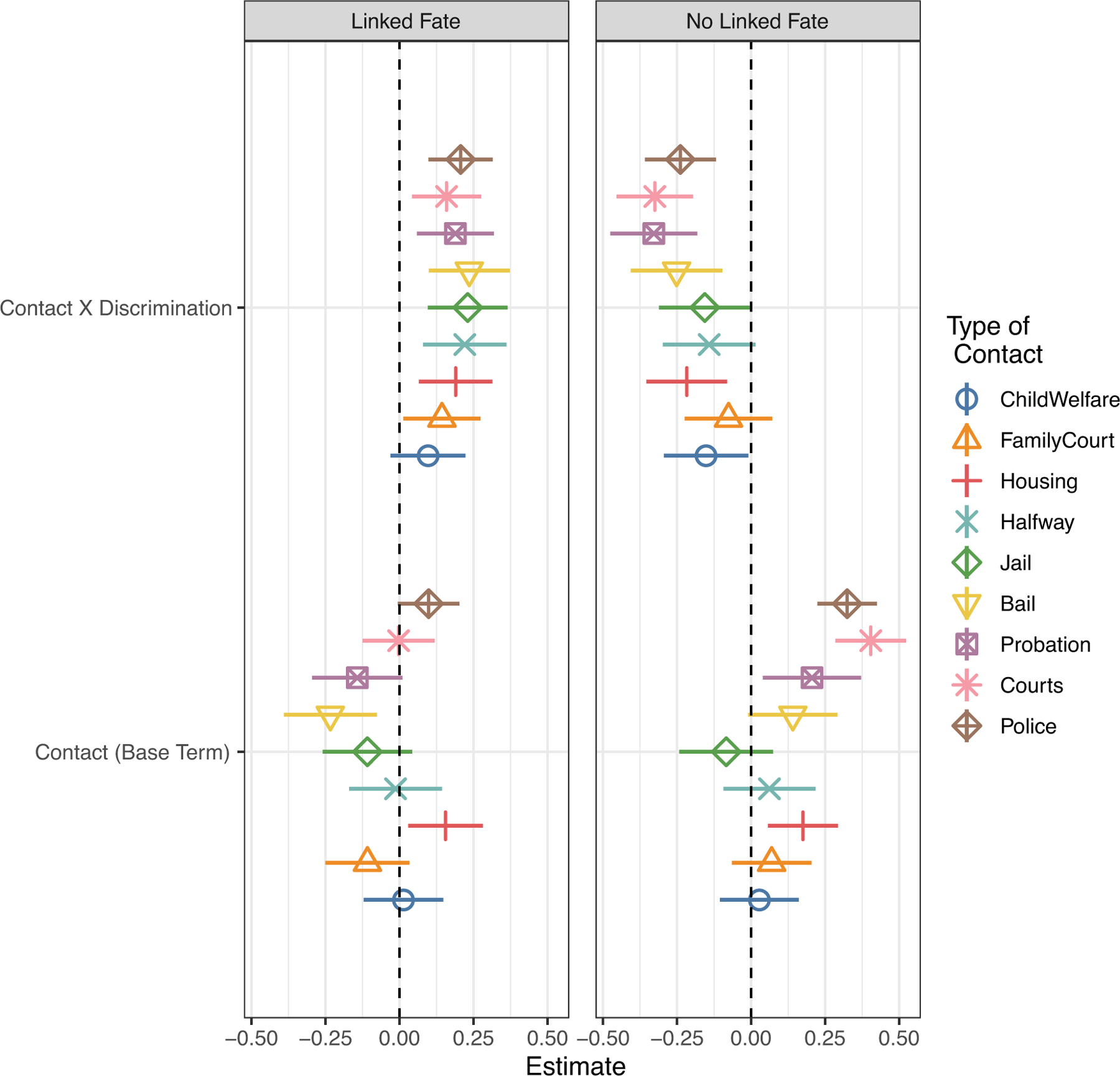
Moderating Effect of a Politicized Group Identity on Involuntary Institutional Contact and Participation among All Respondents in the CMPS *Note*: Model coefficients with 90% confidence bands correspond to regression results reported in online tables A3 and A4.

We argue that accounting for a politicized group identity can help resolve some of these patterns and better explicate the relationship between contact with authoritarian institutions and political engagement. To evaluate our argument, we examine the moderating effect of a politicized group identity and contact on participation. We distinguish between those whose identity is politicized and those whose identity is apolitical by examining the interaction effect of institutional contact and discrimination among subsamples of those with and without linked fate. This empirical strategy yields 18 models among the pooled sample. To render this analysis legible, we display the interaction and base term for contact in figure 3. The full models are reported in online tables A3 and A4.

Figure 3 displays coefficients of each interaction term between contact and discrimination, as well as the coefficients of the base term for contact among those participants who reported linked fate and those who did not. Among those with linked fate (left panel), the interaction between contact and discrimination is positively associated with political engagement across nearly every single measure of institutional contact. This offers support for H1: those who feel a sense of commonality or shared interests with their group and have been targets of discrimination have higher odds of political participation than their counterparts.

In contrast, the coefficients for the moderating effect of discrimination and contact on participation among those with no linked fate (right panel) are largely negative and statistically significant. This suggests that, absent a political connection to one’s group, contact diminishes any differences by perceived discrimination. Most striking about these findings is that they are fairly consistent across all types of institutional contact under study. In an analysis of the base model, without accounting for a politicized identity it is difficult to draw any conclusions about how involuntary contact with different institutions is linked to political engagement. We argued that heightened odds of political engagement are conditional on a politicized group identity and should therefore not vary much by institution type. The general consistency of the findings presented here offer support for this proposition. Interactions with police are consistently associated with higher odds of participation regardless of group identity, suggesting that, in keeping with Schneider and Ingram (1993), interactions with extreme expressions of authoritarian governance can themselves promote political action.

**Figure 4:**
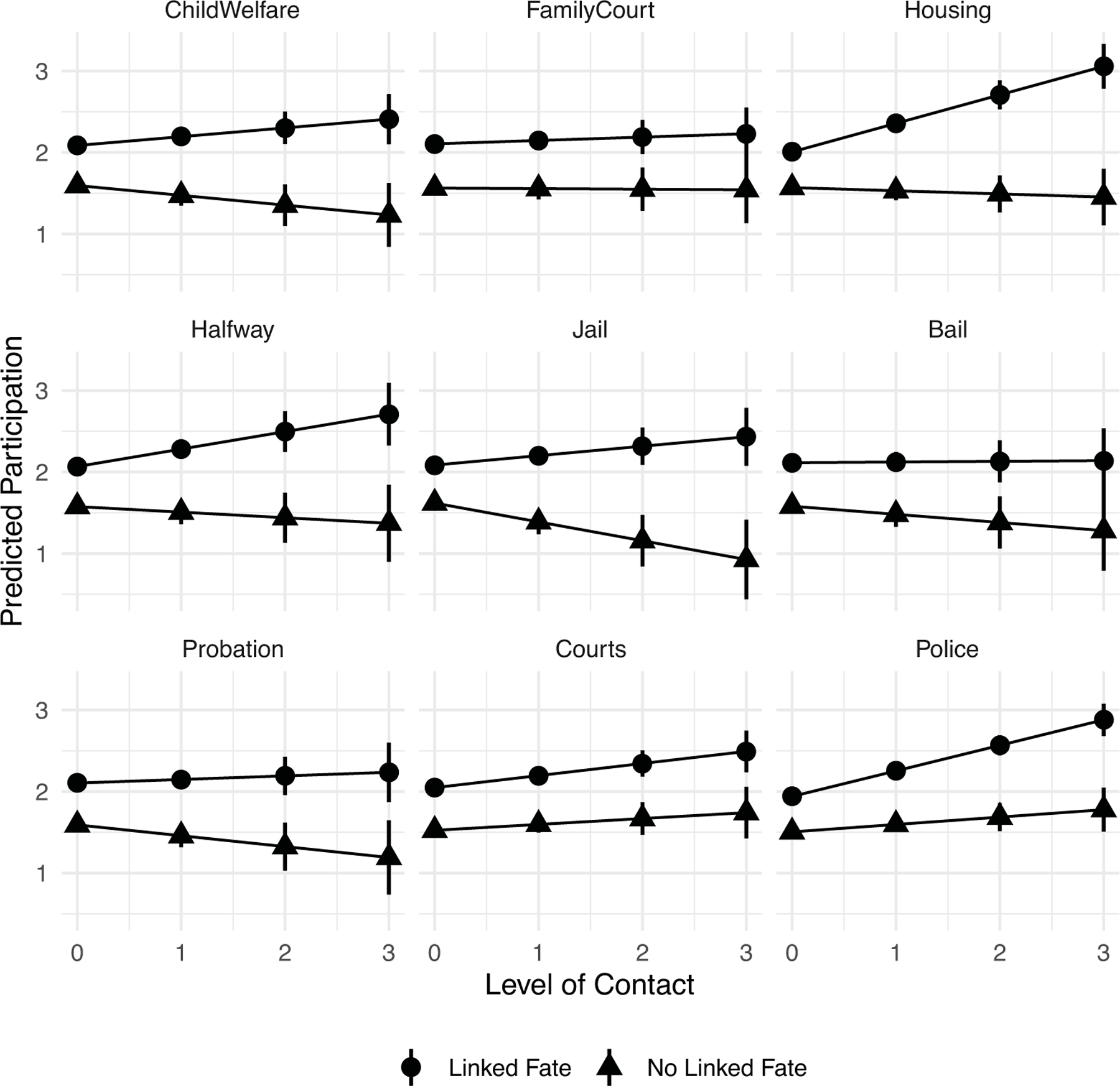
Predicted Value of Participation by Type of Institutional Contact Among Those With and Without a Politicized Group Identity *Note*: Predicted values with 90% confidence bands correspond to regression results reported in online tables A3 and A4. Predicted values are displayed for those who have experienced discrimination but who do and do not have a sense of linked fate.

Because it is difficult to interpret interaction terms by strictly examining coefficients, we further unpack these relationships by calculating the predicted participation score by involuntary contact and reported discrimination among those with or without linked fate. Figure 4 displays these predicted values with 90% confidence bands. Focusing on the top panel (linked fate) we see that individuals with a politicized group identity (circle notation) consistently display higher odds of political participation relative to those who lack such an identity (triangle notation). For instance, looking at those with frequent involuntary police contact (value 3 on the x-axis), we observe a difference of one political act between those with and those without a politicized group identity. Furthermore, the gap in reported political engagement by identity increases across nearly all of the different types of institutions when we compare individuals with no contact (0) to those with frequent contact (3). Overall, the conclusion we draw from the visual depiction of the interaction terms is that respondents with a politicized group identity and repeated involuntary contact report higher levels of political participation compared to their counterparts who lack such an identity but have had frequent contact with authoritarian institutions such as the police.

Recall that the coefficients for the moderating effect of discrimination and contact on participation among those without linked fate were negative and statistically significant (as noted in H2). The bottom panel of figure 4 helps put things in context. The predicted value scores show that, absent any institutional contact (value 0 on the x-axis), those who perceived discrimination display higher odds of political engagement than their counterparts. However, this observed gap by perceived discrimination disappears across all institution types once we look at individuals who reported frequent contact (value 3 on the x-axis). Put differently, among those with frequent contact, the odds of political engagement by perceived discrimination are not statistically different.

Together, the results suggest that involuntary contacts with authoritarian institutions when accompanied by a politicized group identity are generally linked to higher odds of political participation (as hypothesized in H3). Although we observe this general set of patterns across nearly every institution under study, we have not yet examined these relationships among racial subgroups. Our final hypothesis, H4, posits that these relationships do not vary across racial groups. Race structures the likelihood of involuntary interactions with authoritarian institutions, the tenor of those interactions, and the narratives used to connect one’s experiences to a larger group. Yet, existing research suggests that when individuals hold a politicized group identity, that identity should function similarly across groups to convert involuntary experiences with authoritarian institutions into political behavioral outcomes. To assess this claim and following best practices (Masuoka and Junn, 2013), we examine the moderating effect of a politicized group identity on the relationship between involuntary institutional contact and participation among subsamples of Black, Latino, and Asian Americans.

**Figure 5:**
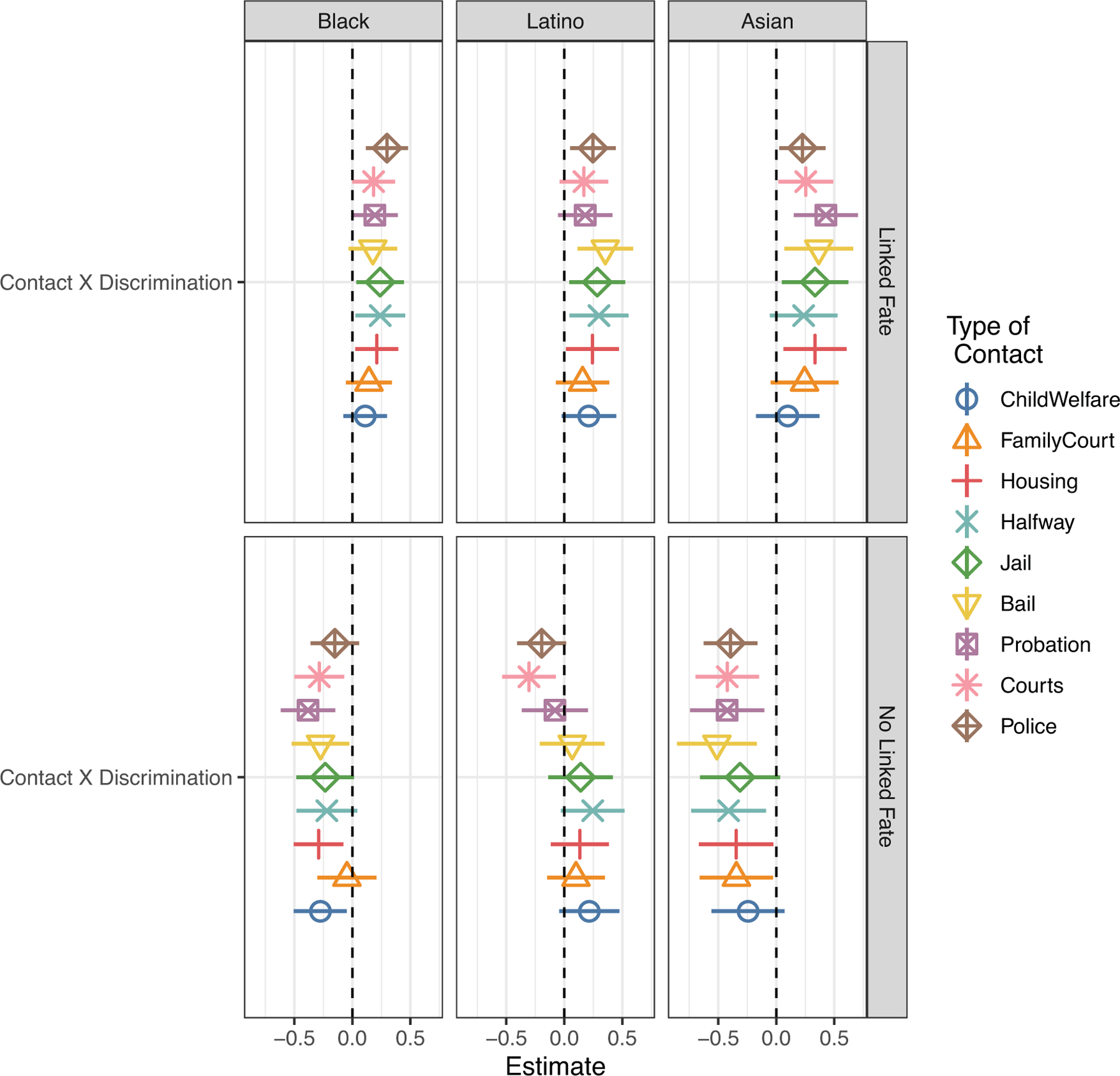
Moderating Effect of a Politicized Group Identity on Involuntary Institutional Contact and Participation by Race *Note*: Predicted values with 90% confidence bands correspond to regression results reported in online tables A5–A10.

The results of this analysis are summarized in figure 5. We provide full models per racial subgroup in online tables A5–A10. Overall, we find generally similar patterns for those with a politicized group identity irrespective of race (top panel in Figure 5). The direction of nearly every contact coefficient is positive and statistically significant despite a reduction in sample size. Conversely, coefficients for contact absent a politicized group identity (bottom panel) are generally negative, although somewhat more mixed among Latinos. Nevertheless, the racial subgroup analyses do not yield findings that are substantively different from the main analysis, particularly when we focus on the relationship between contact and political behavior among those with linked fate. This offers support for H3, which anticipates that a politicized group identity should function to moderate the influence of contact with authoritarian institutions in ways that are similar across subgroups.

## Robustness Checks

On balance, the results presented thus far support our theory, even when we look at racial subgroups. In this section we discuss a set of additional analyses to examine how robust the main results are to alternative modeling strategies and measures. We begin by examining the relationship between institutional contact and group identity for each political participation outcome measure separately. Although we find mixed evidence for this relationship in the literature, it may be the case that individuals affected by authoritarian institutions are especially likely to participate in activities such as protesting or engaging with community-based civic organizations and that one or two items in the index drive the findings overall. Online figure A5 illustrates that this is not the case. Among those with a politicized group identity, different types of institutional contact are positively linked to most of the participation measures. For instance, there are fairly consistent effects in the expected direction for volunteering, contacting government offices, and attending meetings to discuss issues facing the community. With most other outcomes, such as protesting, all of the coefficients are in the expected direction. In the case of protest behavior, only two types of contact are not statistically significant: contact with probation and family court. For only two outcome measures—petition and boycott—the findings are inconclusive with coefficients close to zero.

Some readers may also wonder whether the results would substantively change if we added together all types of authoritarian institutions into a singular index rather than examining them separately. If there is a tipping point whereby chronic contact leads to lower odds of participation, the positive relationship we observe for one institution at a time could be washed out by cumulative contacts. The results of the analysis using a contact index are displayed in online table A11. Here, again, we find support for our theory. Among those with a politicized racial identity, involuntary interactions with authoritarian institutions are positively associated with political participation; absent this identity, involuntary interactions with authoritarian institutions are associated with lower odds of participation. We also see that, with or without linked fate, the contact index is itself associated with increasing levels of participation. Examining the expected values of participation among those with and without a politicized group identity reveals that although contact itself has a slight positive relationship with participation, in the absence of racial linked fate there is no discernible difference between those with and without discrimination, and there is little difference in participation across degrees of institutional contact. Only among those with racial linked fate do we see a clear, strong, positive association between institutional contacts and participation. Although we do not find that repeated contacts are consistently associated with lower levels of participation, as we did in the main body of the analysis, we regard the strong, consistent, and positive relationship between involuntary contacts and participation in the presence of a politicized group identity supportive of our theory.

Finally, some may question the use of racial linked fate to measure a politicized identity across all three racial subgroups. Linked fate specifically operationalizes group consciousness among Black Americans (Sanchez and Vargas 2016). Researchers find that Latinos and Asian Americans are motivated by racial group consciousness under certain circumstances, but findings around linked fate specifically are mixed. The CMPS includes an alternative measure of the importance of a racial group identity for these two groups. Latino and Asian American respondents were asked, “How much is being [Latino/Asian American] an important part of how you see yourself?” Leveraging this measure, we reestimated the moderating effect of a politicized racial identity, substituting identity centrality for linked fate. The results of this analysis are shown in online tables A12 and A13. Generally speaking, the results comport with those derived from models using linked fate. For those with a politicized group identity, involuntary interactions with authoritarian institutions are statistically associated with higher odds of participation across all institutions with the exception of family court and welfare. In these two instances, the coefficients are positive, although they do not achieve statistical significance.

The findings diverge somewhat when we turn to the impact of involuntary interactions with authoritarian institutions absent a politicized group identity (online table A13). Whereas in the main analysis the interaction terms were consistently negative and statistically significant, the findings here are more mixed. Although most of the coefficients are negative, only contact with the courts achieves statistical significance. Moreover, some types of institutional contact independent of either discrimination or a panethnic identity are consistently linked to higher odds of participation. In particular, contact with the police is associated with higher levels of participation, regardless of measures of group identity. This is consistent with the findings displayed in the main analysis, providing evidence for the idea that the police are so threatening that contact spurs participation in its own right.

## Discussion and Conclusion

We began by asking: how do interactions with authoritarian policies shape political engagement? We further asked whether these responses vary by race. Policy feedback theory suggests that policies support participation through democratic structure and benevolent service provision, which enhance civic trust and the resources necessary to politically engage. Policies undercut participation when they are structured in authoritarian ways, conditioning the receipt of goods on behavior, sanctioning access to goods, and, in the most extreme cases, redistributing public and private violence. Such policies, predicated on an individualized logic, obscure the collective nature of public policy, erode trust, and undercut civic capacity. Yet, in a parallel stream of inquiry, racial and ethnic politics scholars demonstrate that sometimes interactions with authoritarian institutions can spur political action. We bring these two literatures into conversation with one another to develop a set of expectations around how and when interactions with authoritarian institutions should heighten or depress the odds of engagement.

We argue and empirically demonstrate that a key factor overlooked by previous research is a politicized group identity. When one’s politicized group identity is salient in the context of an interaction with an authoritarian institution, contact has the potential to mobilize. Viewing one’s experiences with authoritarian policies through the lens of group-based grievances shifts problem solving around those experiences squarely into the public realm. Making sense of one’s experiences through a politicized group identity can suggest an underlying institutional feature that is democratically problematic, and it indicates a group with which to organize for redress. Conversely, we argue that it is in the absence of a politicized group identity that experiences with authoritarian institutions can demoralize, depress, and alienate, pushing individuals away from public life.

To evaluate our argument we used the most comprehensive dataset on racial and ethnic politics currently available: the 2016 CMPS. It includes robust oversamples of each of the largest racial subgroups in the United States. The large sample allows us to slice the data several different ways and to use a combination of moderation and subgroup analysis. Our results support the idea that a politicized group identity conditions participatory responses to contact with a set of related authoritarian policies. These findings hold across a myriad of related institutions among subsamples of Black, Latino, and Asian Americans, and they are robust to a number of alternative specifications.

We ground our insights in the interpretive component of policy feedback theory, which reminds scholars that the lessons learned by interacting with a given policy are conditional on a variety of preexisting analytical frames. Heeding Soss (2005) and Michener (2019), we bring research on the political psychology of race into conversation with work on political learning to chart a clean route to political mobilization. Our primary contribution, then, is a modification to the participatory calculation made under the feedback framework. Our second contribution follows from the first: the logic of our argument derives in part from theorizing around the politics of the criminal justice system, but it invites a cross-institutional comparison, insofar as political psychology marks whether the behavioral outcome will be positive, negative, or null. We therefore extend this mobilizing logic to a class of related policies that Bruch and colleagues (2010) would characterize as authoritarian, arguing that collective disaffection should occur in those instances where individuals decode the liberal-democratic underpinnings of a given policy to reveal (and contest) its ascriptive ends.

Bell (2016) has dubbed this attitude legal estrangement, rebutting the theory and practice of procedural justice that centers compliance with law enforcement as the motivation for repairing degraded relationships with overpoliced communities. Legal estrangement instead turns the focus to what citizens are owed by the criminal justice system in a democratic society, which includes procedural, proximal, and distributional justice. The praxis of procedural justice is individualized, directing focus to the interaction between citizen and state agent; as such it fundamentally undercuts the development of a collective effort to enhance democratic governance. By extending Bell’s (2016) critique to contexts beyond—but often still related to—policing, we join a growing chorus of voices issuing a challenge to the broad class of authoritarian policies identified by Bruch and coauthors (2010). Hyphenate liberalism pervades poverty governance, and racial and ethnic minorities in the United States leverage a politicized group consciousness to contest the quotidian injustices that remind them daily of their second-class status.

Finally, in demonstrating the applicability of our theory across the three largest minoritized groups in the United States, we contribute to the study of racial and ethnic politics. Much of the work on group consciousness and linked fate examines racial subgroups in isolation from one another and in reference to a policy or set of policies that racialize that group in particular. Research on mobilization among Latinos, for example, focuses on the politicizing nature of immigration enforcement by which they are disproportionately affected. Yet, we found that a wide variety of institutions can mobilize individuals with a politicized group identity and that this effect persists across racial subgroups. Although we did not set out to examine how policies can organize an otherwise diverse constituency around a singular cause, our findings do indicate a weakness in racial and ethnic politics research. How might we explain multiracial coalitions mobilized around highly racialized policies like criminal justice and immigration? A policy feedback approach, which centralizes the specifics of how a policy demeans or uplifts citizens, can offer insight into this phenomenon. When citizens make sense of their experiences through a lens that views those experiences as reflective of a larger set of institutional biases, the violation of democratic norms provides the needed catalyst to act.

Our analysis is not without limitations. Although the CMPS permitted us to assess the mobilizing effect of a politicized group consciousness across a variety of authoritarian institutions, we know very little about the nature of those interactions. Our central measure asked respondents how frequently they had involuntary dealings with government agencies. For example, we do not know whether they may have interacted with family court to assist a loved one and so were not personally central to the reason for the hearing. The behavioral consequences of authoritarian policies may vary with the seriousness and intensity of the content of the interaction. We know very little about the contextual factors that may heighten politicized group identity above and beyond the nature of the interaction itself (Nuamah 2020; Nuamah and Ogorzalek 2021), which scholars elsewhere have demonstrated need not be particularly negative to still be racialized (Epp, Haider-Markel, and Maynard-Moody 2014). The primary between-group variation we observe has to do with differences in the likelihood of contact and holding a politicized identity. Beyond this, however, we are unable to explore how different institutions uniquely affect racial subgroups.

## Supplementary Material

Supp Material 2

Supp Material 1

## Figures and Tables

**Table 1:** Distribution of Contact with Authoritarian Institutions in the CMPS

	0	1	2	3
Police	64.86	19.71	11.68	3.75
Courts	70.17	17.47	9.79	2.57
Probation	83.66	7.34	6.75	2.24
Bail	84.80	7.04	6.40	1.77
Halfway	86.04	6.39	5.80	1.78
Housing	81.57	8.45	7.17	2.81
Jail	83.83	7.96	6.28	1.93
Child welfare	82.73	7.76	7.08	2.43
Family	83.20	8.03	6.41	2.36

*Note*: Density distribution come from a question in the 2016 CMPS asking, “How often respondants have you had involuntary contact with each institution.” 0 indicates *never*; 3 indicates *often.*

## Footnotes

1 Bruch, Ferree, and Soss (2010) depart from much feedback research that sorts policies according to whether they are universal or means tested, instead introducing us to the democratic–authoritarian/paternalistic axis. They key in on paternalistic policies, described as authoritarian. We use authoritarian as a shorthand for a range of related policies. We prefer authoritarian to paternalistic because we include criminal justice policies in our analysis, where Bruch, Ferree, and Soss (2010) focus on the administration of antipoverty programs. Paternalism usually refers to the conditioning of the receipt of goods on prosocial behavior, which precludes criminal justice policies that do not deliver a public good to the policy’s targets, instead redistributing state and private violence. The authoritarian descriptor encompasses the broader, though related, scope of programs we study here.

2 Similar findings exist for other groups, such as US Muslims (e.g., Chouhoud, Dana, and Barreto 2019; Jamal 2005).

4 For example, sizable percentages have supported Republican candidates who favor policies that are detrimental to the most marginalized among them, such as restrictive immigration reform.

6 Bruch, Ferree, and Soss (2010) examine TANF, Head Start, and housing assistance but do not analyze differences among racial subgroups. Bruch and Soss (2018) examine the consequences of punitive policies in schools for white Americans, Black Americans, and Latinos. Lerman and Weaver (2014) pay special attention to the racialized nature of the criminal justice system. However, to our knowledge, this is the first study to explicitly compare Black, Latino and Asian Americans and to do so across a class of institutions.

7 The survey had a response rate of 9.9% and a cooperation rate of 57.6%. For more information on the CMPS, see the following: http://www.latinodecisions.com/files/1214/8902/9774/cmps_methodology.pdf.

8 Some may critique the use of general measures of political participation, rather than measuring participation *in speci*fi*c reference to* the policies in question (Campbell 2002). Our decision to examine the impact of authoritarian policies on a wide variety of participatory activities follows several previous feedback studies that evaluated participation in general terms; see Bruch and Soss (2018), Mettler (2002), Soss (1999), and Lerman and Weaver (2014). This is because feedback theory posits that interacting with authoritarian institutions demobilizes by way of diminishing political efficacy and trust, which may lead to declines in engagement with politics altogether.

9 For those who are still interested in seeing the relationship between contact with authoritarian institutions and voting by a politicized group identity, we included figure A4 in the online appendix. We caution against overinterpretation of the findings given limited data points by racial subgroups. As the plot helps illustrate, individuals with a politicized group identity who had contact with varying authoritarian institutions are no more or less likely than their counterparts to report having voted in the 2016 presidential election.

10 For example, children are more likely to be removed to foster care when a parent is imprisoned (Roberts 2014).

11 The correlation matrix for these variables is located in online figure A1.

12 We selected police and child welfare as two examples. The cross tabulations with each of the other eight institutions understudy are is located in online figures A2 and A3.

13 Some may wonder why we use a measure of personal experiences with discrimination, rather than a measure of group discrimination. We do this because measures that ask how much discrimination generally exists toward a group are uninformative as to whether the respondent has personally been treated differently due to social markers. Indeed, research shows that respondents are much more likely to think their group faces discrimination than to report that they have been exposed to discrimination. Previous research has relied on individual-level discrimination measures (Lajevardi 2020; Oskooii 2020; Sanchez 2006; Schildkraut 2005).

14 We do not use a Poisson model, because the events constituting the participation index cannot be assumed to be independent. Moreover, the index is not normally distributed, and the variance is larger than the mean (*μ* = 1.709, *σ*2 = 4.482; Haight 1967). OLS estimators in linear regression will be approximately normally distributed around the true parameter values, which implies that estimated parameters and their confidence interval estimates remain robust (Shao 2003).
