# Supplementary material for: The Participatory Implications of Racialized Policy Feedback
Source: Am Polit Sci Rev. Author manuscript; Available in PMC 2023 Oct 10. (PMC10564389; doi:10.1017/s153759272100311x)
Supplement: Supp Material 2 [file NIHMS1914031-supplement-Supp_Material_2.pdf]

Supplementary Online Appendix

A Description of the Data

Table A1 displays descriptive statistics for every variable employed in the main analysis. The outcome variable (political participation) and the key explanatory variables (contact with various institutions, discrimination and linked fate) are described in the main body of the text. In addition to these variables, all models include controls for political interest, worship attendance, party identification, gender, age, education, income, and race.

Political interest, worship attendance and political efficacy are all understood to positively impact political participation. To measure political interest, we draw on a question that asks respondents: *Some people are very interested in politics while other people can't stand politics, how about you?* Responses are a Likert scale ranging from very interested in politics (coded as 3) to not at all interested in politics (coded as 0). To measure worship attendance respondents were asked: *Do you attend religious service or gathering: at least every week, almost every week, a few times a month, only a few times during the year, hardly ever or never?*. Responses were coded such that 0 indicates those who never attend services, and 5 indicates those who attend every week. We use the question, *How much do you agree or disagree with the statement: Sometimes politics and government seem so complicated that a person like me can't really understand what's going on,* to measure internal efficacy. Response options range from 1 to 5, where 1 indicates that individuals strongly agree with the statement (thus lacking internal efficacy) and 5 indicates individuals strongly disagree with the statement (thus displaying internal efficacy).

Other standard demographic variables include party identification, gender and age. Here, we include a dummy variable for party identification, which indicates whether or not individuals identify with either the democrats or the republicans (coded as 1). Those who do not identify with either party are coded as 0. We code party identification this way because partisans are more likely to participate than non-partisans, but it is not clear that identifying with a specific party should be related to participation writ large or to the likelihood of having contact with the institutions under study. Gender is a dummy variable for female. To measure age, we include dummy variables for the age categories 18-29, 30-39 and 40-64. The comparison category are those over the age of 64. We all age to vary in this fashion since young people are more likely to participate in non-traditional activities like protesting, but individuals who are older are more likely to participate overall.

We also include controls for socioeconomic status, including education and income. Both education and income are expected to positively impact participation and to be negatively associated with the likelihood of having contact with each of the authoritarian institutions under study. Education is a five category variable, where 0 indicates less than high school, 1 indicates high school graduate, 2 indicates some college, 3 indicates college graduates, and 4 indicates that the respondent has completed post-graduate work. To measure income we included dummy variables for those who make less than 40 thousand annually and those who make 40-80 thousand annually, with those in the upper income brackets comprising the comparison category. We arrived at this coding scheme because the survey instrument asked

individuals how much they make annually with more granular income categories, but then follow up with those who at first declined to answer with these broader categories used here. After this follow up question, there is no missing data on income.

B Descriptive Statistics

Table A1: Descriptive statistics of variables used in the CMPS

Statistic	Min	Pctl(25)	Mean	Pctl(75)	Max	St. Dev.
Participation Index	0	0	1.709	3	10	2.241
Police	0	0	0.543	1	3	0.841
Courts	0	0	0.448	1	3	0.773
Probation	0	0	0.276	0	3	0.685
Bail	0	0	0.251	0	3	0.650
Halfway	0	0	0.233	0	3	0.634
Housing	0	0	0.312	0	3	0.726
Jail	0	0	0.263	0	3	0.660
Child Welfare	0	0	0.292	0	3	0.703
Family Court	0	0	0.279	0	3	0.686
Linked Fate	0	0	0.621	1	1	0.485
Discrimination	0	0	0.532	1	1	0.499
Political Efficacy	1	2	2.813	4	5	1.141
Political Interest	0	1	1.757	2	3	0.911
Worship Attendance	0	0	1.859	3	5	1.837
Party ID	0	0	0.662	1	1	0.473
Female	0	0	0.656	1	1	0.475
Age: 18-29	0	0	0.307	1	1	0.461
Age: 30-39	0.	0	0.249	0	1	0.432
Age: 40-64	0.	0	0.368	1	1	0.482
Education	0	1	2.269	3	4	1.141
Income: <40k	0	0	0.422	1	1	0.494
Income: 40-79k	0	0	0.309	1	1	0.462
Black	0	0	0.340	1	1	0.474
Latino	0	0	0.330	1	1	0.470
Asian	0	0	0.330	1	1	0.470

Figure A1: **The Correlation Between Types of Institutional Contact**

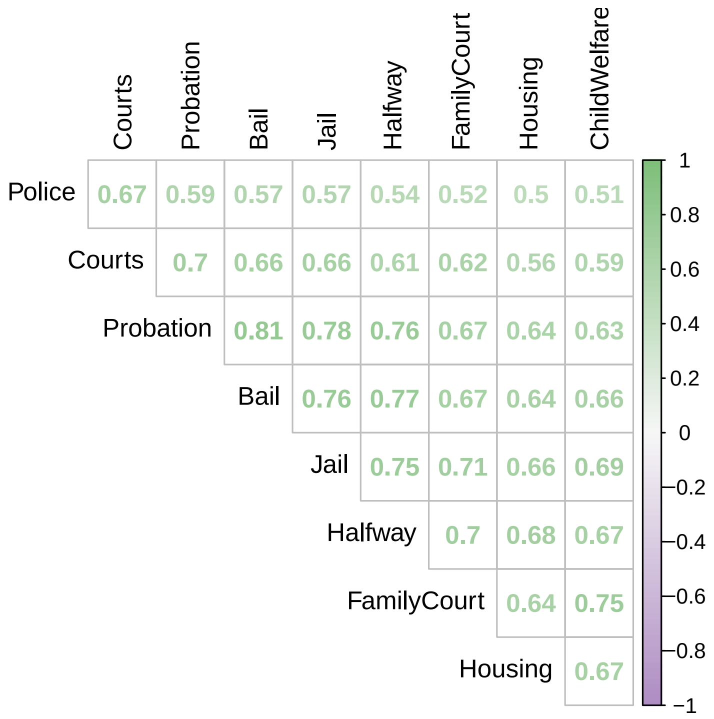

Figure A2:

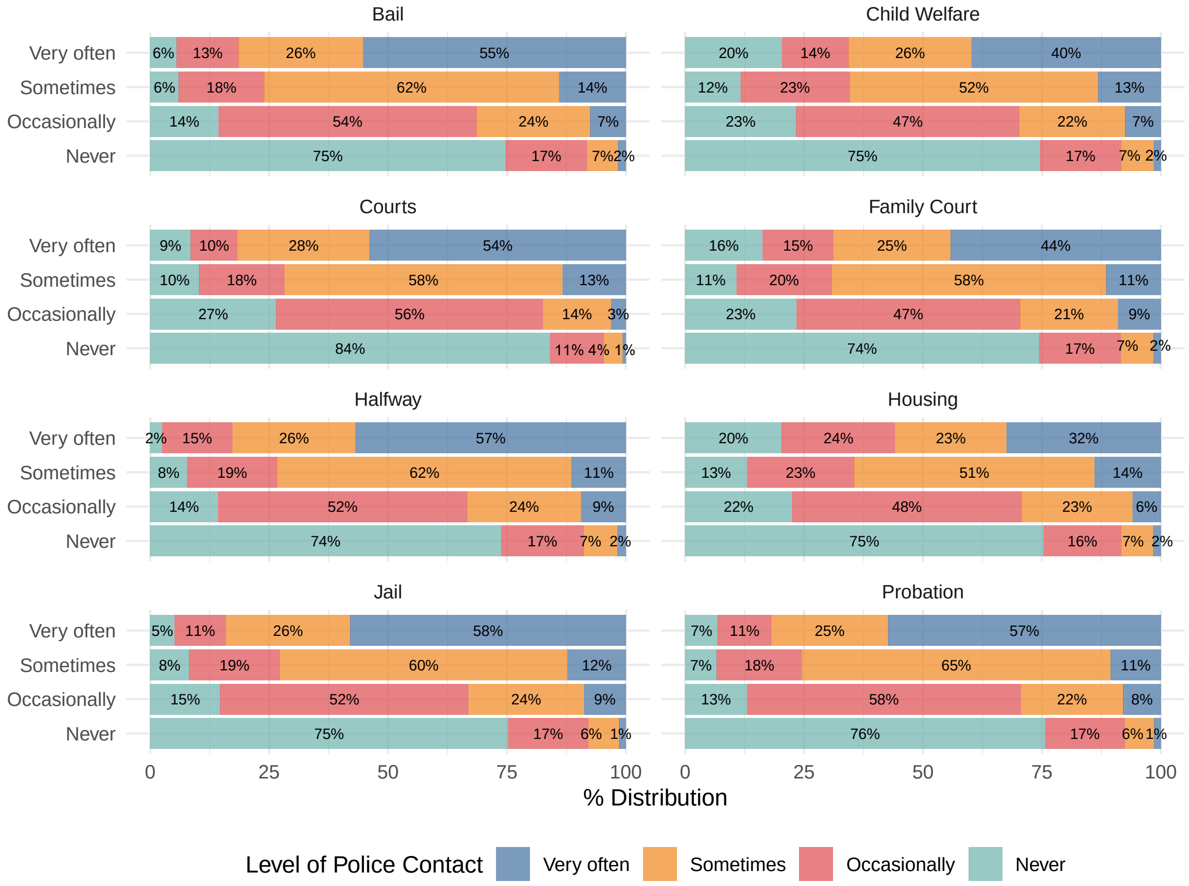
Bail
Child Welfare
Courts
Family Court
Halfway
Housing
Jail
Probation
Very often
Sometimes
Occasionally
Never
% Distribution
Level of Police Contact
Very often
Sometimes
Occasionally
Never

Figure A3:

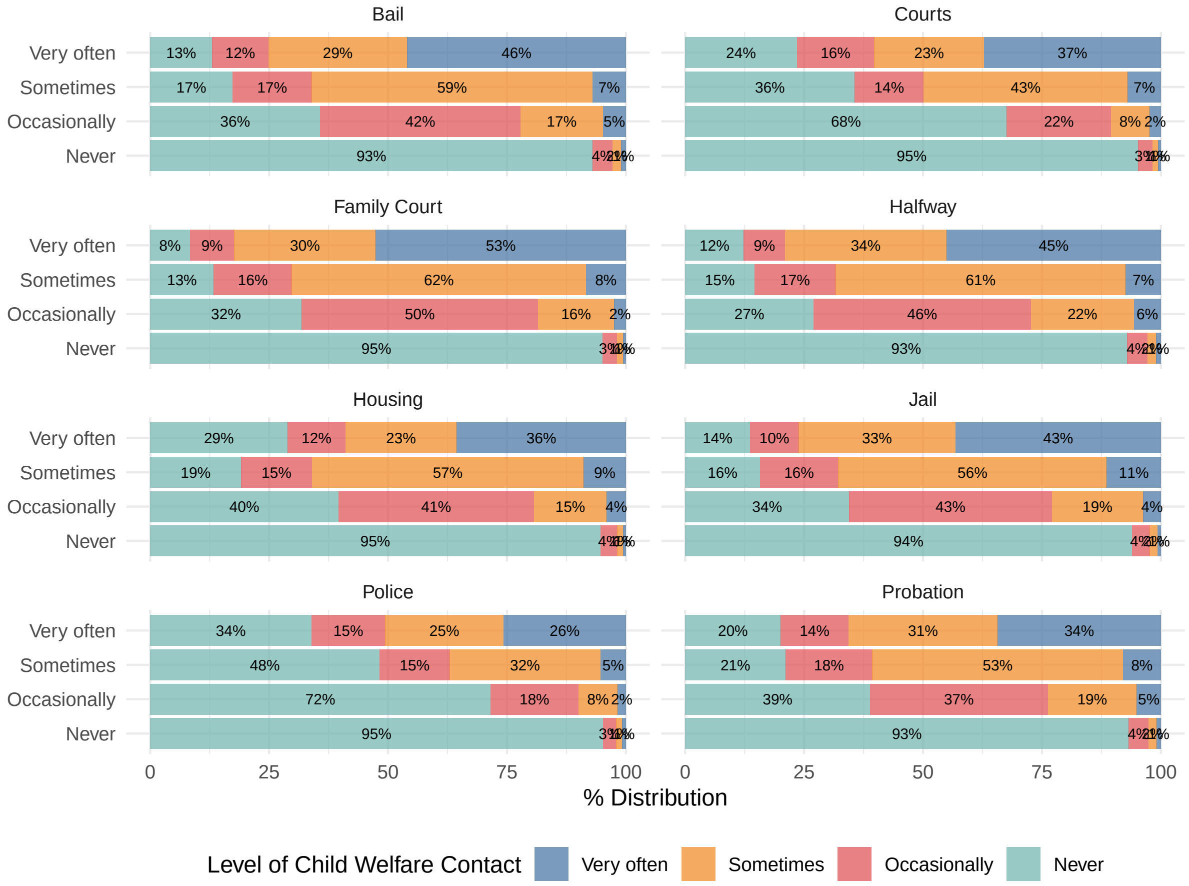

Bail
Courts
Family Court
Halfway
Housing
Jail
Police
Probation
Very often
Sometimes
Occasionally
Never
Bail — Very often: 13% 12% 29% 46%
Bail — Sometimes: 17% 17% 59% 7%
Bail — Occasionally: 36% 42% 17% 5%
Bail — Never: 93%
Courts — Very often: 24% 16% 23% 37%
Courts — Sometimes: 36% 14% 43% 7%
Courts — Occasionally: 68% 22% 8% 2%
Courts — Never: 95%
Family Court — Very often: 8% 9% 30% 53%
Family Court — Sometimes: 13% 16% 62% 8%
Family Court — Occasionally: 32% 50% 16% 2%
Family Court — Never: 95%
Halfway — Very often: 12% 9% 34% 45%
Halfway — Sometimes: 15% 17% 61% 7%
Halfway — Occasionally: 27% 46% 22% 6%
Halfway — Never: 93%
Housing — Very often: 29% 12% 23% 36%
Housing — Sometimes: 19% 15% 57% 9%
Housing — Occasionally: 40% 41% 15% 4%
Housing — Never: 95%
Jail — Very often: 14% 10% 33% 43%
Jail — Sometimes: 16% 16% 56% 11%
Jail — Occasionally: 34% 43% 19% 4%
Jail — Never: 94%
Police — Very often: 34% 15% 25% 26%
Police — Sometimes: 48% 15% 32% 5%
Police — Occasionally: 72% 18% 8% 2%
Police — Never: 95%
Probation — Very often: 20% 14% 31% 34%
Probation — Sometimes: 21% 18% 53% 8%
Probation — Occasionally: 39% 37% 19% 5%
Probation — Never: 93%
0 25 50 75 100
% Distribution
Level of Child Welfare Contact
Very often
Sometimes
Occasionally
Never

C Tables Associated with The Main Analyses

Table A2: The Impact of Institutional Contact on Political Participation

	Dependent variable:
	Political Participation
Child Welfare	0.047
	(0.050)
Family Court	0.001
	(0.052)
Housing	0.213^{***}
	(0.044)
Halfway	0.090
	(0.061)
Jail	−0.017
	(0.059)
Bail	−0.039
	(0.062)
Probation	−0.008
	(0.060)
Courts	0.152^{***}
	(0.043)
Police	0.239^{***}
	(0.035)
Discrimination	0.528^{***}
	(0.043)
Linked Fate	0.431^{***}
	(0.044)
Political Efficacy	0.089^{***}
	(0.019)
Political Interest	0.734^{***}
	(0.025)
Worship Attendance	0.058^{***}
	(0.012)
Party ID	0.015
	(0.045)
Female	−0.006
	(0.045)
Age 18-29	-0.143^{*}
	(0.087)
Age 30-39	-0.333^{***}
	(0.086)
Age 40-64	-0.348^{***}
	(0.082)
Education	0.173^{***}
	(0.022)
Income < 40K	-0.433^{***}
	(0.059)
Income 40-70K	−0.063
	(0.056)
Asian	-0.252^{***}
	(0.056)
Latino	0.099^{*}
	(0.052)
Constant	-0.663^{***}
	(0.138)
Observations	9,098
R^2	0.243
Adjusted R^2	0.241
Note:	*p<0.1; **p<0.05; ***p<0.01

Table A3: Moderation analysis: The impact of discrimination and institutional contact on participation, among those with linked fate

	Dependent variable:								
	Political Participation								
	Bail	Court	Family	Halfway	Housing	Jail	Police	Probation	Child Welfare
	(1)	(2)	(3)	(4)	(5)	(6)	(7)	(8)	(9)
Contact	−0.234**	−0.003	−0.108	−0.013	0.155**	−0.109	0.098	−0.142	0.013
	(0.098)	(0.076)	(0.089)	(0.098)	(0.079)	(0.094)	(0.065)	(0.095)	(0.084)
Discrimination	0.526***	0.519***	0.549***	0.535***	0.529***	0.526***	0.478***	0.535***	0.562***
	(0.064)	(0.068)	(0.064)	(0.063)	(0.065)	(0.064)	(0.070)	(0.064)	(0.064)
Child Welfare	0.079	0.080	0.082	0.083	0.082	0.084	0.081	0.081	
	(0.065)	(0.065)	(0.065)	(0.065)	(0.065)	(0.065)	(0.065)	(0.065)	
Family Court	−0.008	−0.012		−0.007	−0.013	−0.011	−0.009	−0.010	−0.009
	(0.069)	(0.069)		(0.069)	(0.069)	(0.069)	(0.069)	(0.069)	(0.069)
Housing	0.291***	0.288***	0.286***	0.290***		0.291***	0.293***	0.290***	0.287***
	(0.058)	(0.058)	(0.058)	(0.058)		(0.058)	(0.058)	(0.058)	(0.058)
Halfway	0.127	0.132*	0.136*		0.137*	0.136*	0.136*	0.127	0.133*
	(0.080)	(0.080)	(0.080)		(0.080)	(0.080)	(0.080)	(0.080)	(0.080)
Jail	0.039	0.046	0.047	0.046	0.050		0.043	0.040	0.049
	(0.076)	(0.076)	(0.076)	(0.076)	(0.076)		(0.076)	(0.076)	(0.076)
Bail		−0.081	−0.086	−0.088	−0.086	−0.088	−0.085	−0.079	−0.090
		(0.083)	(0.083)	(0.083)	(0.083)	(0.083)	(0.083)	(0.083)	(0.083)
Probation	−0.015	−0.016	−0.018	−0.023	−0.017	−0.023	−0.014		−0.017
	(0.078)	(0.078)	(0.078)	(0.078)	(0.078)	(0.078)	(0.078)		(0.078)
Courts	0.108*		0.105*	0.107*	0.105*	0.108*	0.105*	0.107*	0.105*
	(0.057)		(0.057)	(0.057)	(0.057)	(0.057)	(0.057)	(0.057)	(0.057)
Police	0.242***	0.241***	0.243***	0.244***	0.245***	0.242***		0.243***	0.242***
	(0.045)	(0.045)	(0.045)	(0.045)	(0.045)	(0.045)		(0.045)	(0.045)
Political Efficacy	0.105***	0.105***	0.104***	0.105***	0.104***	0.104***	0.106***	0.105***	0.103***
	(0.026)	(0.026)	(0.026)	(0.026)	(0.026)	(0.026)	(0.026)	(0.026)	(0.026)
Political Interest	0.884***	0.883***	0.883***	0.883***	0.884***	0.884***	0.884***	0.884***	0.884***
	(0.035)	(0.035)	(0.035)	(0.035)	(0.035)	(0.035)	(0.035)	(0.035)	(0.035)
Worship Attendance	0.049***	0.050***	0.049***	0.049***	0.049***	0.049***	0.051***	0.050***	0.050***
	(0.016)	(0.016)	(0.016)	(0.016)	(0.016)	(0.016)	(0.016)	(0.016)	(0.016)
Party ID	0.094	0.092	0.094	0.093	0.092	0.093	0.093	0.093	0.094
	(0.062)	(0.062)	(0.062)	(0.062)	(0.062)	(0.062)	(0.062)	(0.062)	(0.062)
Female	0.027	0.027	0.026	0.026	0.026	0.028	0.028	0.026	0.026
	(0.061)	(0.061)	(0.061)	(0.061)	(0.061)	(0.061)	(0.061)	(0.061)	(0.061)
Age 40-64	−0.172	−0.170	−0.175	−0.172	−0.173	−0.175	−0.172	−0.173	−0.174
	(0.122)	(0.122)	(0.122)	(0.122)	(0.122)	(0.122)	(0.122)	(0.122)	(0.123)
Age 30-39	−0.347***	−0.346***	−0.350***	−0.349***	−0.349***	−0.348***	−0.345***	−0.348***	−0.348***
	(0.122)	(0.122)	(0.123)	(0.122)	(0.122)	(0.122)	(0.122)	(0.122)	(0.123)
Age 18-29	−0.498***	−0.495***	−0.500***	−0.499***	−0.497***	−0.498***	−0.491***	−0.499***	−0.498***
	(0.117)	(0.117)	(0.117)	(0.117)	(0.117)	(0.117)	(0.117)	(0.117)	(0.117)
Education	0.221***	0.219***	0.220***	0.220***	0.219***	0.220***	0.217***	0.220***	0.219***
	(0.030)	(0.030)	(0.030)	(0.030)	(0.030)	(0.030)	(0.030)	(0.030)	(0.030)
Income < 40K	−0.566***	−0.566***	−0.563***	−0.565***	−0.567***	−0.569***	−0.569***	−0.567***	−0.563***
	(0.081)	(0.081)	(0.081)	(0.081)	(0.081)	(0.081)	(0.081)	(0.081)	(0.081)
Income 40-70K	−0.156**	−0.155**	−0.152**	−0.156**	−0.155**	−0.157**	−0.155**	−0.155**	−0.151**
	(0.076)	(0.076)	(0.076)	(0.076)	(0.076)	(0.076)	(0.076)	(0.076)	(0.076)
Asian	−0.361***	−0.356***	−0.355***	−0.358***	−0.357***	−0.361***	−0.349***	−0.358***	−0.355***
	(0.075)	(0.075)	(0.075)	(0.075)	(0.075)	(0.075)	(0.075)	(0.075)	(0.075)
Latino	0.174**	0.177**	0.180**	0.174**	0.175**	0.174**	0.179**	0.175**	0.178**
	(0.071)	(0.071)	(0.071)	(0.071)	(0.071)	(0.071)	(0.070)	(0.071)	(0.071)
Contact X Disc	0.236***	0.159**	0.143*	0.220**	0.190**	0.230***	0.206***	0.189**	0.096
	(0.085)	(0.073)	(0.081)	(0.088)	(0.078)	(0.084)	(0.068)	(0.081)	(0.079)
Constant	−0.582***	−0.576***	−0.593***	−0.583***	−0.577***	−0.574***	−0.560***	−0.585***	−0.599***
	(0.192)	(0.193)	(0.193)	(0.192)	(0.193)	(0.193)	(0.193)	(0.193)	(0.193)
Observations	5,658	5,658	5,658	5,658	5,658	5,658	5,658	5,658	5,658
R^2	0.245	0.245	0.245	0.245	0.245	0.245	0.246	0.245	0.245
Adjusted R^2	0.242	0.242	0.242	0.242	0.242	0.242	0.242	0.242	0.241
Note:									*p<0.1; **p<0.05; ***p<0.01

Table A4: Moderation analysis: The impact of discrimination and institutional contact on participation, among those without linked fate

	Dependent variable:								
	Political Participation								
	Bail	Court	Family	Halfway	Housing	Jail	Police	Probation	Child Welfare
	(1)	(2)	(3)	(4)	(5)	(6)	(7)	(8)	(9)
Contact	0.141	0.404***	0.069	0.062	0.175**	−0.084	0.325***	0.206**	0.028
	(0.094)	(0.074)	(0.084)	(0.097)	(0.074)	(0.099)	(0.063)	(0.104)	(0.083)
Discrimination	0.448***	0.517***	0.413***	0.424***	0.455***	0.429***	0.500***	0.472***	0.433***
	(0.062)	(0.066)	(0.063)	(0.062)	(0.063)	(0.062)	(0.067)	(0.062)	(0.063)
Child Welfare	−0.036	−0.047	−0.037	−0.036	−0.039	−0.038	−0.035	−0.031	
	(0.073)	(0.073)	(0.073)	(0.073)	(0.073)	(0.073)	(0.073)	(0.073)	
Family Court	0.038	0.026		0.034	0.037	0.034	0.022	0.032	0.038
	(0.075)	(0.075)		(0.075)	(0.075)	(0.075)	(0.075)	(0.075)	(0.075)
Housing	0.066	0.058	0.074	0.070		0.068	0.062	0.061	0.071
	(0.063)	(0.063)	(0.063)	(0.063)		(0.063)	(0.063)	(0.063)	(0.063)
Halfway	0.004	0.017	0.003		−0.001	0.014	0.009	0.007	0.006
	(0.088)	(0.088)	(0.088)		(0.088)	(0.088)	(0.088)	(0.088)	(0.088)
Jail	−0.144	−0.147	−0.147	−0.138	−0.155*		−0.154*	−0.149	−0.150
	(0.092)	(0.091)	(0.092)	(0.092)	(0.092)		(0.092)	(0.092)	(0.092)
Bail		0.037	0.056	0.048	0.045	0.050	0.049	0.033	0.050
		(0.089)	(0.089)	(0.089)	(0.089)	(0.089)	(0.089)	(0.089)	(0.089)
Probation	0.052	0.051	0.043	0.044	0.051	0.047	0.039		0.051
	(0.093)	(0.093)	(0.093)	(0.093)	(0.093)	(0.093)	(0.093)		(0.093)
Courts	0.249***		0.245***	0.248***	0.245***	0.249***	0.255***	0.254***	0.242***
	(0.063)		(0.063)	(0.063)	(0.063)	(0.063)	(0.063)	(0.063)	(0.063)
Police	0.207***	0.209***	0.209***	0.208***	0.205***	0.206***		0.200***	0.208***
	(0.052)	(0.052)	(0.052)	(0.052)	(0.052)	(0.052)		(0.052)	(0.052)
Political Efficacy	0.060**	0.060**	0.058**	0.059**	0.060**	0.059**	0.060**	0.060**	0.059**
	(0.025)	(0.025)	(0.025)	(0.026)	(0.025)	(0.026)	(0.025)	(0.025)	(0.025)
Political Interest	0.532***	0.531***	0.534***	0.533***	0.531***	0.533***	0.530***	0.531***	0.533***
	(0.032)	(0.032)	(0.032)	(0.032)	(0.032)	(0.032)	(0.032)	(0.032)	(0.032)
Worship Attendance	0.072***	0.071***	0.073***	0.073***	0.072***	0.072***	0.071***	0.071***	0.072***
	(0.016)	(0.016)	(0.016)	(0.016)	(0.016)	(0.016)	(0.016)	(0.016)	(0.016)
Party ID	−0.078	−0.073	−0.077	−0.079	−0.076	−0.078	−0.080	−0.084	−0.078
	(0.060)	(0.060)	(0.060)	(0.060)	(0.060)	(0.060)	(0.060)	(0.060)	(0.060)
Female	−0.071	−0.074	−0.077	−0.075	−0.074	−0.076	−0.075	−0.072	−0.076
	(0.061)	(0.061)	(0.061)	(0.061)	(0.061)	(0.061)	(0.061)	(0.061)	(0.061)
Age 40-64	−0.119	−0.124	−0.122	−0.123	−0.123	−0.122	−0.122	−0.123	−0.121
	(0.113)	(0.113)	(0.113)	(0.113)	(0.113)	(0.113)	(0.113)	(0.113)	(0.113)
Age 30-39	−0.356***	−0.352***	−0.357***	−0.359***	−0.361***	−0.356***	−0.351***	−0.354***	−0.358***
	(0.111)	(0.111)	(0.111)	(0.111)	(0.111)	(0.111)	(0.111)	(0.111)	(0.111)
Age 18-29	−0.134	−0.135	−0.136	−0.137	−0.136	−0.135	−0.133	−0.133	−0.137
	(0.105)	(0.105)	(0.105)	(0.105)	(0.105)	(0.105)	(0.105)	(0.105)	(0.105)
Education	0.099***	0.100***	0.100***	0.100***	0.099***	0.099***	0.101***	0.099***	0.100***
	(0.028)	(0.028)	(0.028)	(0.028)	(0.028)	(0.028)	(0.028)	(0.028)	(0.028)
Income < 40K	−0.243***	−0.240***	−0.241***	−0.239***	−0.239***	−0.241***	−0.243***	−0.242***	−0.242***
	(0.082)	(0.082)	(0.082)	(0.082)	(0.082)	(0.082)	(0.082)	(0.082)	(0.082)
Income 40-70K	0.063	0.062	0.061	0.062	0.062	0.062	0.060	0.065	0.061
	(0.077)	(0.076)	(0.077)	(0.077)	(0.077)	(0.077)	(0.076)	(0.076)	(0.077)
Asian	−0.133*	−0.140*	−0.131*	−0.129*	−0.131*	−0.131*	−0.139*	−0.139*	−0.132*
	(0.078)	(0.078)	(0.078)	(0.078)	(0.078)	(0.078)	(0.078)	(0.078)	(0.078)
Latino	−0.024	−0.032	−0.023	−0.021	−0.024	−0.024	−0.030	−0.032	−0.025
	(0.071)	(0.071)	(0.071)	(0.071)	(0.071)	(0.071)	(0.071)	(0.071)	(0.071)
Contact X Disc	−0.252***	−0.325***	−0.076	−0.141	−0.217**	−0.156	−0.239***	−0.328***	−0.152*
	(0.097)	(0.081)	(0.092)	(0.098)	(0.085)	(0.097)	(0.075)	(0.092)	(0.089)
Constant	−0.143	−0.162	−0.126	−0.134	−0.143	−0.132	−0.153	−0.141	−0.133
	(0.183)	(0.183)	(0.183)	(0.183)	(0.183)	(0.183)	(0.183)	(0.183)	(0.183)
Observations	3,442	3,442	3,442	3,442	3,442	3,442	3,442	3,442	3,442
R^2	0.192	0.194	0.190	0.191	0.192	0.191	0.193	0.193	0.191
Adjusted R^2	0.186	0.188	0.185	0.185	0.186	0.185	0.187	0.188	0.185

Note: *p<0.1; **p<0.05; ***p<0.01

Table A5: Moderation analysis: The impact of discrimination and institutional contact on participation, among Black Americans with linked fate

	Dependent variable:								
	Political Participation								
	Bail	Court	Family	Halfway	Housing	Jail	Police	Probation	Child Welfare
	(1)	(2)	(3)	(4)	(5)	(6)	(7)	(8)	(9)
Contact	−0.200	0.080	0.001	−0.037	0.124	−0.007	−0.125	−0.218	−0.029
	(0.149)	(0.122)	(0.134)	(0.145)	(0.114)	(0.143)	(0.111)	(0.142)	(0.123)
Discrimination	0.426***	0.384***	0.434***	0.406***	0.391***	0.393***	0.295**	0.410***	0.440***
	(0.117)	(0.127)	(0.118)	(0.116)	(0.120)	(0.118)	(0.130)	(0.118)	(0.119)
Police	0.096	0.093	0.099	0.100	0.101	0.100		0.099	0.099
	(0.070)	(0.070)	(0.070)	(0.070)	(0.070)	(0.070)		(0.070)	(0.070)
Courts	0.212**		0.206**	0.212**	0.210**	0.212**	0.201**	0.210**	0.207**
	(0.087)		(0.087)	(0.087)	(0.086)	(0.086)	(0.086)	(0.087)	(0.087)
Probation	−0.080	−0.083	−0.086	−0.092	−0.089	−0.091	−0.077		−0.086
	(0.112)	(0.112)	(0.112)	(0.112)	(0.112)	(0.112)	(0.112)		(0.112)
Bail		−0.081	−0.089	−0.096	−0.091	−0.093	−0.091	−0.076	−0.094
		(0.123)	(0.123)	(0.123)	(0.123)	(0.123)	(0.123)	(0.123)	(0.123)
Halfway	0.114	0.122	0.124		0.129	0.127	0.130	0.116	0.122
	(0.114)	(0.114)	(0.115)		(0.114)	(0.114)	(0.114)	(0.114)	(0.114)
Housing	0.272***	0.274***	0.269***	0.273***		0.274***	0.277***	0.272***	0.270***
	(0.076)	(0.076)	(0.076)	(0.076)		(0.076)	(0.076)	(0.076)	(0.076)
Jail	0.149	0.158	0.157	0.160	0.163		0.164	0.149	0.159
	(0.112)	(0.112)	(0.112)	(0.112)	(0.112)		(0.112)	(0.112)	(0.113)
Child Welfare	0.049	0.050	0.054	0.050	0.052	0.055	0.058	0.051	
	(0.090)	(0.090)	(0.090)	(0.090)	(0.090)	(0.090)	(0.090)	(0.090)	
Family Court	0.102	0.096		0.102	0.098	0.099	0.101	0.100	0.103
	(0.096)	(0.096)		(0.096)	(0.096)	(0.096)	(0.096)	(0.096)	(0.096)
Political Efficacy	0.066	0.065	0.064	0.066*	0.066	0.065	0.068*	0.066	0.064
	(0.040)	(0.040)	(0.040)	(0.040)	(0.040)	(0.040)	(0.040)	(0.040)	(0.040)
Political Interest	0.804***	0.804***	0.803***	0.805***	0.806***	0.804***	0.805***	0.805***	0.805***
	(0.057)	(0.057)	(0.057)	(0.057)	(0.057)	(0.057)	(0.057)	(0.057)	(0.057)
Worship Attendance	0.098***	0.099***	0.097***	0.097***	0.098***	0.098***	0.100***	0.098***	0.097***
	(0.026)	(0.026)	(0.026)	(0.026)	(0.026)	(0.026)	(0.026)	(0.026)	(0.026)
Party ID	0.037	0.036	0.032	0.038	0.030	0.036	0.038	0.035	0.033
	(0.111)	(0.111)	(0.111)	(0.111)	(0.111)	(0.111)	(0.111)	(0.111)	(0.111)
Female	0.058	0.057	0.059	0.059	0.058	0.064	0.062	0.059	0.058
	(0.104)	(0.104)	(0.104)	(0.104)	(0.104)	(0.104)	(0.104)	(0.104)	(0.104)
Age 18-29	−0.259	−0.257	−0.266	−0.256	−0.262	−0.268	−0.271	−0.263	−0.262
	(0.184)	(0.184)	(0.185)	(0.184)	(0.184)	(0.184)	(0.184)	(0.184)	(0.184)
Age 30-39	−0.510***	−0.510***	−0.515***	−0.510***	−0.514***	−0.514***	−0.507***	−0.512***	−0.513***
	(0.184)	(0.184)	(0.184)	(0.184)	(0.184)	(0.184)	(0.184)	(0.184)	(0.184)
Age 40-64	−0.677***	−0.676***	−0.681***	−0.679***	−0.677***	−0.683***	−0.676***	−0.680***	−0.680***
	(0.167)	(0.167)	(0.168)	(0.167)	(0.167)	(0.167)	(0.167)	(0.167)	(0.168)
Education	0.261***	0.258***	0.261***	0.261***	0.258***	0.259***	0.257***	0.260***	0.260***
	(0.051)	(0.051)	(0.051)	(0.051)	(0.051)	(0.051)	(0.051)	(0.051)	(0.051)
Income < 40K	−0.602***	−0.605***	−0.603***	−0.600***	−0.605***	−0.609***	−0.611***	−0.605***	−0.603***
	(0.142)	(0.142)	(0.142)	(0.142)	(0.142)	(0.142)	(0.142)	(0.142)	(0.142)
Income 40-70K	−0.138	−0.137	−0.138	−0.141	−0.140	−0.144	−0.148	−0.140	−0.137
	(0.141)	(0.141)	(0.141)	(0.141)	(0.141)	(0.141)	(0.141)	(0.141)	(0.141)
Contact X Disc	0.167	0.174	0.133	0.237*	0.199*	0.233*	0.290**	0.185	0.107
	(0.132)	(0.116)	(0.124)	(0.134)	(0.116)	(0.128)	(0.114)	(0.124)	(0.118)
Constant	−0.256	−0.219	−0.247	−0.246	−0.222	−0.223	−0.169	−0.243	−0.251
	(0.305)	(0.307)	(0.306)	(0.305)	(0.306)	(0.306)	(0.307)	(0.305)	(0.306)
Observations	2,076	2,076	2,076	2,076	2,076	2,076	2,076	2,076	2,076
R^2	0.248	0.249	0.248	0.249	0.249	0.249	0.250	0.249	0.248
Adjusted R^2	0.240	0.241	0.240	0.241	0.241	0.241	0.242	0.241	0.240
Note:									*p<0.1; **p<0.05; ***p<0.01

Table A6: Moderation analysis: The impact of discrimination and institutional contact on participation, among Black Americans without linked fate

	Dependent variable:								
	Political Participation								
	Bail	Court	Family	Halfway	Housing	Jail	Police	Probation	Child Welfare
	(1)	(2)	(3)	(4)	(5)	(6)	(7)	(8)	(9)
Contact	0.255	0.429^{***}	0.170	−0.041	0.187^{*}	−0.030	0.241^{**}	0.302^{*}	0.086
	(0.156)	(0.134)	(0.150)	(0.165)	(0.112)	(0.158)	(0.117)	(0.170)	(0.136)
Discrimination	0.399^{***}	0.454^{***}	0.325^{**}	0.375^{***}	0.440^{***}	0.393^{***}	0.396^{***}	0.448^{***}	0.419^{***}
	(0.126)	(0.134)	(0.129)	(0.125)	(0.130)	(0.127)	(0.138)	(0.127)	(0.129)
Police	0.145	0.141	0.149^{*}	0.144	0.142	0.134		0.125	0.146
	(0.089)	(0.089)	(0.090)	(0.090)	(0.089)	(0.090)		(0.090)	(0.089)
Courts	0.266^{**}		0.260^{**}	0.267^{**}	0.265^{**}	0.265^{**}	0.264^{**}	0.276^{**}	0.254^{**}
	(0.109)		(0.109)	(0.109)	(0.109)	(0.109)	(0.109)	(0.109)	(0.109)
Probation	0.069	0.074	0.054	0.064	0.070	0.069	0.049		0.074
	(0.141)	(0.141)	(0.141)	(0.141)	(0.140)	(0.141)	(0.140)		(0.141)
Bail		0.113	0.129	0.103	0.102	0.127	0.130	0.098	0.108
		(0.142)	(0.143)	(0.144)	(0.143)	(0.142)	(0.142)	(0.142)	(0.143)
Halfway	−0.170	−0.141	−0.155		−0.155	−0.134	−0.154	−0.153	−0.140
	(0.143)	(0.143)	(0.144)		(0.143)	(0.144)	(0.143)	(0.143)	(0.143)
Housing	0.022	0.025	0.033	0.025		0.021	0.029	0.019	0.033
	(0.089)	(0.088)	(0.089)	(0.089)		(0.089)	(0.089)	(0.088)	(0.088)
Jail	−0.129	−0.159	−0.146	−0.131	−0.169		−0.158	−0.151	−0.167
	(0.142)	(0.142)	(0.143)	(0.143)	(0.143)		(0.143)	(0.142)	(0.143)
Child Welfare	−0.058	−0.065	−0.057	−0.054	−0.044	−0.062	−0.052	−0.058	
	(0.114)	(0.114)	(0.115)	(0.114)	(0.114)	(0.114)	(0.114)	(0.114)	
Family Court	0.137	0.128		0.146	0.146	0.135	0.130	0.141	0.151
	(0.126)	(0.126)		(0.126)	(0.125)	(0.126)	(0.126)	(0.125)	(0.126)
Political Efficacy	0.008	0.006	0.004	0.007	0.007	0.008	0.004	0.008	0.007
	(0.048)	(0.048)	(0.048)	(0.048)	(0.048)	(0.048)	(0.048)	(0.048)	(0.048)
Political Interest	0.554^{***}	0.553^{***}	0.555^{***}	0.554^{***}	0.555^{***}	0.553^{***}	0.555^{***}	0.553^{***}	0.557^{***}
	(0.062)	(0.062)	(0.062)	(0.062)	(0.062)	(0.062)	(0.062)	(0.062)	(0.062)
Worship Attendance	0.057^{*}	0.057^{*}	0.058^{*}	0.058^{*}	0.056^{*}	0.058^{*}	0.057^{*}	0.055^{*}	0.058^{*}
	(0.031)	(0.031)	(0.031)	(0.031)	(0.031)	(0.031)	(0.031)	(0.031)	(0.031)
Party ID	−0.067	−0.070	−0.063	−0.063	−0.057	−0.061	−0.072	−0.079	−0.059
	(0.128)	(0.128)	(0.128)	(0.128)	(0.128)	(0.128)	(0.128)	(0.128)	(0.128)
Female	−0.031	−0.038	−0.047	−0.037	−0.037	−0.038	−0.044	−0.029	−0.037
	(0.126)	(0.126)	(0.126)	(0.126)	(0.126)	(0.126)	(0.126)	(0.126)	(0.126)
Age 18-29	-0.585^{***}	-0.601^{***}	-0.593^{***}	-0.594^{***}	-0.591^{***}	-0.586^{***}	-0.592^{***}	-0.588^{***}	-0.586^{***}
	(0.223)	(0.222)	(0.223)	(0.223)	(0.222)	(0.223)	(0.223)	(0.222)	(0.223)
Age 30-39	-0.856^{***}	-0.866^{***}	-0.866^{***}	-0.866^{***}	-0.861^{***}	-0.856^{***}	-0.863^{***}	-0.853^{***}	-0.858^{***}
	(0.222)	(0.222)	(0.222)	(0.222)	(0.222)	(0.222)	(0.222)	(0.222)	(0.222)
Age 40-64	-0.707^{***}	-0.721^{***}	-0.714^{***}	-0.714^{***}	-0.710^{***}	-0.711^{***}	-0.715^{***}	-0.704^{***}	-0.721^{***}
	(0.204)	(0.204)	(0.204)	(0.204)	(0.203)	(0.204)	(0.204)	(0.203)	(0.204)
Education	0.147^{**}	0.151^{**}	0.156^{***}	0.152^{**}	0.144^{**}	0.148^{**}	0.155^{***}	0.145^{**}	0.148^{**}
	(0.059)	(0.059)	(0.059)	(0.059)	(0.059)	(0.059)	(0.059)	(0.059)	(0.059)
Income < 40K	-0.408^{**}	-0.399^{**}	-0.403^{**}	-0.402^{**}	-0.407^{**}	-0.406^{**}	-0.401^{**}	-0.408^{**}	-0.412^{**}
	(0.178)	(0.178)	(0.178)	(0.178)	(0.178)	(0.178)	(0.178)	(0.177)	(0.178)
Income 40-70K	−0.026	−0.023	−0.037	−0.031	−0.035	−0.030	−0.031	−0.012	−0.040
	(0.181)	(0.181)	(0.181)	(0.181)	(0.181)	(0.181)	(0.181)	(0.181)	(0.181)
Contact X Disc	-0.287^{*}	-0.286^{**}	−0.055	−0.234	-0.299^{**}	−0.247	−0.154	-0.386^{***}	-0.283^{**}
	(0.155)	(0.134)	(0.159)	(0.163)	(0.134)	(0.155)	(0.131)	(0.147)	(0.143)
Constant	0.529	0.522	0.569	0.536	0.519	0.535	0.543	0.520	0.525
	(0.359)	(0.359)	(0.359)	(0.360)	(0.359)	(0.359)	(0.360)	(0.358)	(0.359)
Observations	1,020	1,020	1,020	1,020	1,020	1,020	1,020	1,020	1,020
R^2	0.203	0.204	0.200	0.202	0.204	0.202	0.201	0.206	0.203
Adjusted R^2	0.185	0.186	0.183	0.184	0.186	0.185	0.184	0.188	0.186
Note:									*p<0.1; **p<0.05; ***p<0.01

Table A7: Moderation analysis: The impact of discrimination and institutional contact on participation, among Latinos with linked fate

	Dependent variable:								
	Political Participation								
	Bail	Court	Family	Halfway	Housing	Jail	Police	Probation	Child Welfare
	(1)	(2)	(3)	(4)	(5)	(6)	(7)	(8)	(9)
Contact	−0.325**	−0.068	−0.063	−0.150	0.118	−0.175	0.175	−0.206	−0.072
	(0.163)	(0.133)	(0.149)	(0.177)	(0.146)	(0.164)	(0.117)	(0.167)	(0.151)
Discrimination	0.549***	0.575***	0.615***	0.588***	0.581***	0.578***	0.520***	0.606***	0.602***
	(0.119)	(0.128)	(0.119)	(0.117)	(0.120)	(0.119)	(0.131)	(0.119)	(0.120)
Police	0.351***	0.346***	0.346***	0.349***	0.350***	0.346***		0.348***	0.345***
	(0.083)	(0.083)	(0.083)	(0.083)	(0.083)	(0.083)		(0.083)	(0.083)
Courts	0.050		0.048	0.047	0.046	0.052	0.054	0.049	0.051
	(0.103)		(0.103)	(0.103)	(0.103)	(0.103)	(0.103)	(0.103)	(0.103)
Probation	−0.099	−0.087	−0.089	−0.096	−0.085	−0.101	−0.085		−0.095
	(0.135)	(0.135)	(0.135)	(0.135)	(0.135)	(0.135)	(0.135)		(0.135)
Bail		−0.111	−0.115	−0.117	−0.114	−0.114	−0.107	−0.112	−0.115
		(0.140)	(0.140)	(0.140)	(0.140)	(0.140)	(0.140)	(0.140)	(0.140)
Halfway	0.025	0.038	0.045		0.042	0.045	0.040	0.035	0.052
	(0.143)	(0.143)	(0.143)		(0.143)	(0.143)	(0.143)	(0.143)	(0.143)
Housing	0.291***	0.287***	0.283**	0.287***		0.292***	0.295***	0.292***	0.287***
	(0.110)	(0.110)	(0.110)	(0.110)		(0.110)	(0.110)	(0.110)	(0.110)
Jail	0.004	0.014	0.018	0.017	0.019		0.009	0.009	0.016
	(0.131)	(0.131)	(0.131)	(0.131)	(0.131)		(0.131)	(0.131)	(0.131)
Child Welfare	0.061	0.060	0.061	0.070	0.066	0.061	0.058	0.058	
	(0.117)	(0.117)	(0.117)	(0.117)	(0.117)	(0.117)	(0.117)	(0.117)	
Family Court	0.044	0.036		0.042	0.033	0.043	0.039	0.038	0.040
	(0.122)	(0.122)		(0.122)	(0.122)	(0.122)	(0.122)	(0.122)	(0.122)
Political Efficacy	0.119**	0.121**	0.120**	0.119**	0.118**	0.119**	0.120**	0.120**	0.119**
	(0.049)	(0.049)	(0.049)	(0.049)	(0.049)	(0.049)	(0.049)	(0.049)	(0.049)
Political Interest	0.902***	0.902***	0.903***	0.900***	0.903***	0.903***	0.903***	0.902***	0.904***
	(0.066)	(0.066)	(0.066)	(0.066)	(0.066)	(0.066)	(0.066)	(0.066)	(0.066)
Worship Attendance	0.018	0.019	0.019	0.019	0.019	0.019	0.020	0.020	0.019
	(0.031)	(0.031)	(0.031)	(0.031)	(0.031)	(0.031)	(0.031)	(0.031)	(0.031)
Party ID	0.006	0.007	0.010	0.007	0.005	0.006	0.011	0.008	0.007
	(0.116)	(0.117)	(0.117)	(0.116)	(0.117)	(0.116)	(0.116)	(0.117)	(0.117)
Female	−0.007	−0.003	−0.005	−0.006	−0.007	−0.006	−0.004	−0.009	−0.003
	(0.117)	(0.118)	(0.118)	(0.118)	(0.118)	(0.118)	(0.118)	(0.118)	(0.118)
Age 18-29	0.073	0.060	0.070	0.060	0.064	0.067	0.058	0.062	0.066
	(0.349)	(0.349)	(0.349)	(0.349)	(0.349)	(0.349)	(0.349)	(0.349)	(0.349)
Age 30-39	0.085	0.071	0.080	0.070	0.075	0.079	0.069	0.072	0.079
	(0.352)	(0.352)	(0.352)	(0.352)	(0.352)	(0.352)	(0.352)	(0.352)	(0.352)
Age 40-64	−0.032	−0.041	−0.029	−0.041	−0.036	−0.034	−0.041	−0.039	−0.031
	(0.347)	(0.348)	(0.347)	(0.347)	(0.347)	(0.347)	(0.347)	(0.348)	(0.347)
Education	0.270***	0.264***	0.265***	0.265***	0.264***	0.265***	0.262***	0.266***	0.264***
	(0.056)	(0.056)	(0.056)	(0.056)	(0.056)	(0.056)	(0.056)	(0.056)	(0.056)
Income < 40K	−0.784***	−0.772***	−0.771***	−0.783***	−0.782***	−0.783***	−0.781***	−0.776***	−0.772***
	(0.159)	(0.159)	(0.159)	(0.159)	(0.159)	(0.159)	(0.159)	(0.159)	(0.159)
Income 40-70K	−0.176	−0.169	−0.166	−0.176	−0.170	−0.174	−0.167	−0.168	−0.166
	(0.157)	(0.157)	(0.157)	(0.157)	(0.157)	(0.157)	(0.157)	(0.157)	(0.157)
Contact X Disc	0.370**	0.185	0.160	0.293*	0.254*	0.292*	0.252**	0.188	0.199
	(0.151)	(0.132)	(0.144)	(0.160)	(0.144)	(0.151)	(0.123)	(0.147)	(0.148)
Constant	−0.671	−0.681	−0.715	−0.668	−0.673	−0.678	−0.644	−0.696	−0.703
	(0.448)	(0.450)	(0.449)	(0.449)	(0.449)	(0.449)	(0.450)	(0.449)	(0.449)
Observations	1,735	1,735	1,735	1,735	1,735	1,735	1,735	1,735	1,735
R^2	0.258	0.256	0.256	0.257	0.257	0.257	0.257	0.256	0.256
Adjusted R^2	0.248	0.247	0.246	0.247	0.247	0.247	0.248	0.247	0.247
Note:									*p<0.1; **p<0.05; ***p<0.01

Table A8: Moderation analysis: The impact of discrimination and institutional contact on participation, among Latinos without linked fate

	Dependent variable:								
	Political Participation								
	Bail	Court	Family	Halfway	Housing	Jail	Police	Probation	Child Welfare
	(1)	(2)	(3)	(4)	(5)	(6)	(7)	(8)	(9)
Contact	−0.082	0.541***	−0.071	−0.092	−0.032	−0.102	0.219**	0.027	−0.115
	(0.167)	(0.126)	(0.128)	(0.160)	(0.139)	(0.167)	(0.104)	(0.182)	(0.137)
Discrimination	0.426***	0.548***	0.413***	0.391***	0.405***	0.410***	0.518***	0.455***	0.389***
	(0.104)	(0.110)	(0.105)	(0.103)	(0.105)	(0.104)	(0.112)	(0.104)	(0.105)
Police	0.143	0.140	0.147	0.149*	0.147	0.144		0.141	0.148*
	(0.089)	(0.089)	(0.090)	(0.089)	(0.089)	(0.089)		(0.089)	(0.089)
Courts	0.400***		0.399***	0.395***	0.401***	0.395***	0.408***	0.399***	0.405***
	(0.107)		(0.107)	(0.107)	(0.107)	(0.107)	(0.107)	(0.107)	(0.107)
Probation	0.007	−0.033	0.008	0.022	0.005	0.018	−0.015		−0.002
	(0.176)	(0.176)	(0.176)	(0.176)	(0.176)	(0.176)	(0.176)		(0.176)
Bail		−0.062	−0.071	−0.077	−0.062	−0.057	−0.054	−0.057	−0.071
		(0.156)	(0.157)	(0.156)	(0.156)	(0.156)	(0.156)	(0.156)	(0.156)
Halfway	−0.006	0.016	0.003		0.002	−0.010	0.001	−0.001	0.002
	(0.148)	(0.148)	(0.148)		(0.148)	(0.148)	(0.148)	(0.148)	(0.148)
Housing	0.029	0.006	0.033	0.037		0.034	0.013	0.026	0.038
	(0.122)	(0.122)	(0.123)	(0.122)		(0.122)	(0.122)	(0.122)	(0.122)
Jail	−0.046	−0.036	−0.051	−0.056	−0.045		−0.044	−0.055	−0.051
	(0.157)	(0.156)	(0.156)	(0.156)	(0.156)		(0.156)	(0.156)	(0.156)
Child Welfare	−0.044	−0.060	−0.043	−0.043	−0.039	−0.046	−0.046	−0.040	
	(0.126)	(0.126)	(0.126)	(0.126)	(0.126)	(0.126)	(0.126)	(0.127)	
Family Court	−0.035	−0.035		−0.027	−0.031	−0.033	−0.042	−0.031	−0.036
	(0.115)	(0.114)		(0.115)	(0.115)	(0.115)	(0.115)	(0.115)	(0.114)
Political Efficacy	0.097**	0.097**	0.097**	0.097**	0.097**	0.097**	0.100**	0.097**	0.097**
	(0.042)	(0.042)	(0.042)	(0.042)	(0.042)	(0.042)	(0.042)	(0.042)	(0.042)
Political Interest	0.502***	0.494***	0.503***	0.504***	0.504***	0.503***	0.494***	0.499***	0.503***
	(0.054)	(0.053)	(0.054)	(0.053)	(0.054)	(0.054)	(0.054)	(0.054)	(0.053)
Worship Attendance	0.065**	0.063**	0.065**	0.064**	0.065**	0.065**	0.063**	0.065**	0.066**
	(0.026)	(0.026)	(0.026)	(0.026)	(0.026)	(0.026)	(0.026)	(0.026)	(0.026)
Party ID	−0.088	−0.076	−0.090	−0.085	−0.089	−0.087	−0.081	−0.087	−0.089
	(0.099)	(0.099)	(0.099)	(0.099)	(0.099)	(0.099)	(0.099)	(0.099)	(0.099)
Female	−0.158	−0.160	−0.155	−0.155	−0.156	−0.156	−0.161	−0.157	−0.156
	(0.104)	(0.103)	(0.104)	(0.103)	(0.103)	(0.103)	(0.103)	(0.103)	(0.103)
Age 18-29	0.146	0.143	0.143	0.149	0.148	0.149	0.146	0.146	0.142
	(0.200)	(0.200)	(0.200)	(0.200)	(0.200)	(0.200)	(0.200)	(0.200)	(0.200)
Age 30-39	−0.167	−0.165	−0.169	−0.163	−0.164	−0.169	−0.161	−0.167	−0.168
	(0.199)	(0.199)	(0.199)	(0.199)	(0.199)	(0.199)	(0.199)	(0.199)	(0.199)
Age 40-64	0.185	0.186	0.185	0.191	0.188	0.186	0.191	0.184	0.186
	(0.188)	(0.188)	(0.188)	(0.188)	(0.188)	(0.188)	(0.188)	(0.188)	(0.188)
Education	0.165***	0.163***	0.166***	0.165***	0.164***	0.166***	0.164***	0.165***	0.166***
	(0.046)	(0.046)	(0.046)	(0.046)	(0.046)	(0.046)	(0.046)	(0.046)	(0.046)
Income < 40K	−0.230*	−0.237*	−0.226	−0.233*	−0.232*	−0.228*	−0.238*	−0.232*	−0.224
	(0.137)	(0.137)	(0.138)	(0.137)	(0.137)	(0.137)	(0.137)	(0.137)	(0.137)
Income 40-70K	0.105	0.095	0.109	0.107	0.104	0.107	0.094	0.101	0.110
	(0.133)	(0.133)	(0.133)	(0.133)	(0.133)	(0.133)	(0.133)	(0.133)	(0.133)
Contact X Disc	0.065	−0.305**	0.105	0.252	0.139	0.144	−0.192	−0.080	0.216
	(0.175)	(0.144)	(0.156)	(0.172)	(0.157)	(0.174)	(0.132)	(0.178)	(0.162)
Constant	−0.520*	−0.538*	−0.523*	−0.519*	−0.516*	−0.522*	−0.536*	−0.522*	−0.519*
	(0.295)	(0.294)	(0.295)	(0.295)	(0.295)	(0.295)	(0.295)	(0.295)	(0.295)
Observations	1,264	1,264	1,264	1,264	1,264	1,264	1,264	1,264	1,264
R^2	0.196	0.198	0.196	0.197	0.196	0.196	0.197	0.196	0.197
Adjusted R^2	0.181	0.184	0.182	0.183	0.182	0.182	0.183	0.181	0.182
Note:									*p<0.1; **p<0.05; ***p<0.01

Table A9: Moderation analysis: The impact of discrimination and institutional contact on participation, among Asians with linked fate

	Dependent variable:								
	Political Participation								
	Bail	Court	Family	Halfway	Housing	Jail	Police	Probation	Child Welfare
	(1)	(2)	(3)	(4)	(5)	(6)	(7)	(8)	(9)
Contact	−0.139	−0.211	−0.823***	−0.001	0.348*	−0.372*	0.263**	0.294	0.073
	(0.249)	(0.153)	(0.218)	(0.221)	(0.192)	(0.216)	(0.113)	(0.229)	(0.207)
Discrimination	0.571***	0.555***	0.587***	0.588***	0.568***	0.574***	0.535***	0.555***	0.608***
	(0.098)	(0.103)	(0.098)	(0.098)	(0.098)	(0.098)	(0.107)	(0.098)	(0.098)
Police	0.391***	0.394***	0.389***	0.389***	0.394***	0.386***		0.388***	0.387***
	(0.088)	(0.088)	(0.088)	(0.088)	(0.088)	(0.088)		(0.088)	(0.088)
Courts	−0.056		−0.055	−0.056	−0.066	−0.059	−0.058	−0.055	−0.060
	(0.119)		(0.119)	(0.119)	(0.119)	(0.119)	(0.119)	(0.119)	(0.119)
Probation	0.526**	0.531**	0.526**	0.509**	0.524**	0.536**	0.518**		0.515**
	(0.212)	(0.212)	(0.212)	(0.211)	(0.212)	(0.212)	(0.211)		(0.212)
Bail		0.090	0.086	0.112	0.086	0.086	0.093	0.077	0.094
		(0.215)	(0.215)	(0.215)	(0.215)	(0.215)	(0.215)	(0.215)	(0.215)
Halfway	0.182	0.157	0.157		0.161	0.144	0.156	0.150	0.137
	(0.196)	(0.195)	(0.196)		(0.195)	(0.195)	(0.195)	(0.195)	(0.195)
Housing	0.525***	0.508***	0.517***	0.523***		0.529***	0.525***	0.529***	0.510***
	(0.172)	(0.172)	(0.172)	(0.172)		(0.172)	(0.172)	(0.172)	(0.172)
Jail	−0.175	−0.170	−0.171	−0.169	−0.165		−0.177	−0.174	−0.151
	(0.177)	(0.178)	(0.178)	(0.178)	(0.177)		(0.178)	(0.177)	(0.177)
Child Welfare	0.094	0.103	0.099	0.109	0.099	0.120	0.103	0.104	
	(0.181)	(0.181)	(0.181)	(0.180)	(0.181)	(0.180)	(0.180)	(0.180)	
Family Court	−0.690***	−0.673***		−0.686***	−0.696***	−0.704***	−0.683***	−0.688***	−0.693***
	(0.189)	(0.189)		(0.189)	(0.189)	(0.189)	(0.189)	(0.189)	(0.190)
Political Efficacy	0.151***	0.153***	0.150***	0.152***	0.151***	0.150***	0.153***	0.152***	0.150***
	(0.046)	(0.046)	(0.046)	(0.046)	(0.046)	(0.046)	(0.046)	(0.046)	(0.046)
Political Interest	0.915***	0.916***	0.918***	0.916***	0.916***	0.917***	0.918***	0.915***	0.917***
	(0.061)	(0.061)	(0.061)	(0.061)	(0.061)	(0.061)	(0.061)	(0.061)	(0.061)
Worship Attendance	−0.003	−0.002	−0.002	−0.002	−0.003	−0.002	−0.001	−0.002	−0.002
	(0.026)	(0.026)	(0.026)	(0.026)	(0.026)	(0.026)	(0.026)	(0.026)	(0.026)
Party ID	0.182*	0.183*	0.189*	0.185*	0.187*	0.186*	0.182*	0.183*	0.190*
	(0.098)	(0.098)	(0.098)	(0.098)	(0.097)	(0.098)	(0.098)	(0.097)	(0.098)
Female	0.034	0.035	0.031	0.033	0.034	0.032	0.035	0.032	0.034
	(0.097)	(0.097)	(0.097)	(0.097)	(0.097)	(0.097)	(0.097)	(0.097)	(0.097)
Age 18-29	−0.179	−0.172	−0.180	−0.180	−0.180	−0.180	−0.172	−0.174	−0.180
	(0.190)	(0.191)	(0.191)	(0.191)	(0.190)	(0.190)	(0.191)	(0.190)	(0.191)
Age 30-39	−0.337*	−0.326*	−0.333*	−0.335*	−0.338*	−0.331*	−0.322*	−0.332*	−0.329*
	(0.186)	(0.186)	(0.186)	(0.186)	(0.186)	(0.186)	(0.186)	(0.186)	(0.186)
Age 40-64	−0.494***	−0.485***	−0.492***	−0.495***	−0.493***	−0.490***	−0.477***	−0.490***	−0.495***
	(0.181)	(0.181)	(0.181)	(0.181)	(0.181)	(0.181)	(0.181)	(0.180)	(0.181)
Education	0.121**	0.119**	0.119**	0.119**	0.120**	0.120**	0.118**	0.121**	0.118**
	(0.050)	(0.050)	(0.050)	(0.050)	(0.050)	(0.050)	(0.050)	(0.050)	(0.050)
Income < 40K	−0.217*	−0.218*	−0.211*	−0.214*	−0.215*	−0.216*	−0.212*	−0.221*	−0.212*
	(0.128)	(0.128)	(0.128)	(0.128)	(0.128)	(0.128)	(0.128)	(0.128)	(0.128)
Income 40-70K	−0.229**	−0.231**	−0.225**	−0.225**	−0.229**	−0.229**	−0.222**	−0.233**	−0.222**
	(0.110)	(0.110)	(0.110)	(0.110)	(0.110)	(0.110)	(0.110)	(0.110)	(0.110)
Contact X Disc	0.351*	0.232	0.230	0.230	0.314*	0.322*	0.215*	0.414**	0.082
	(0.186)	(0.148)	(0.183)	(0.183)	(0.171)	(0.179)	(0.125)	(0.173)	(0.171)
Constant	−0.929***	−0.931***	−0.941***	−0.939***	−0.928***	−0.932***	−0.930***	−0.926***	−0.951***
	(0.293)	(0.293)	(0.293)	(0.293)	(0.293)	(0.293)	(0.293)	(0.293)	(0.293)
Observations	1,847	1,847	1,847	1,847	1,847	1,847	1,847	1,847	1,847
R^2	0.247	0.247	0.247	0.247	0.247	0.247	0.247	0.248	0.246
Adjusted R^2	0.238	0.238	0.238	0.238	0.238	0.238	0.238	0.239	0.237

Note: *p<0.1; **p<0.05; ***p<0.01

Table A10: Moderation analysis: The impact of discrimination and institutional contact on participation, among Asians without linked fate

	Dependent variable:								
	Political Participation								
	Bail	Court	Family	Halfway	Housing	Jail	Police	Probation	Child Welfare
	(1)	(2)	(3)	(4)	(5)	(6)	(7)	(8)	(9)
Contact	−0.016	0.181	0.183	0.532**	0.439**	−0.338	0.486***	0.334	0.149
	(0.190)	(0.130)	(0.209)	(0.215)	(0.175)	(0.235)	(0.111)	(0.219)	(0.192)
Discrimination	0.484***	0.525***	0.470***	0.477***	0.473***	0.457***	0.550***	0.480***	0.455***
	(0.099)	(0.105)	(0.100)	(0.099)	(0.100)	(0.099)	(0.107)	(0.099)	(0.100)
Police	0.315***	0.347***	0.325***	0.326***	0.324***	0.322***		0.327***	0.327***
	(0.095)	(0.095)	(0.095)	(0.095)	(0.095)	(0.095)		(0.095)	(0.095)
Courts	0.036		0.035	0.030	0.029	0.033	0.057	0.031	0.035
	(0.118)		(0.118)	(0.118)	(0.118)	(0.118)	(0.118)	(0.118)	(0.118)
Probation	0.186	0.168	0.177	0.194	0.150	0.202	0.178		0.164
	(0.204)	(0.203)	(0.204)	(0.204)	(0.203)	(0.206)	(0.203)		(0.204)
Bail		−0.126	−0.129	−0.150	−0.098	−0.122	−0.158	−0.158	−0.111
		(0.189)	(0.190)	(0.191)	(0.189)	(0.190)	(0.190)	(0.191)	(0.190)
Halfway	0.375*	0.377*	0.365*		0.387*	0.376*	0.391**	0.393**	0.376*
	(0.198)	(0.198)	(0.198)		(0.199)	(0.198)	(0.198)	(0.198)	(0.199)
Housing	0.357**	0.304*	0.334**	0.323*		0.333**	0.305*	0.305*	0.311*
	(0.167)	(0.168)	(0.167)	(0.167)		(0.167)	(0.168)	(0.168)	(0.169)
Jail	−0.394*	−0.436*	−0.448**	−0.402*	−0.417*		−0.438*	−0.367	−0.438*
	(0.225)	(0.224)	(0.224)	(0.225)	(0.225)		(0.224)	(0.227)	(0.225)
Child Welfare	0.007	0.022	0.019	0.032	0.006	0.024	0.020	0.017	
	(0.172)	(0.171)	(0.172)	(0.171)	(0.172)	(0.172)	(0.171)	(0.172)	
Family Court	0.027	0.041		0.004	0.034	0.024	0.028	0.011	0.020
	(0.192)	(0.192)		(0.193)	(0.192)	(0.193)	(0.192)	(0.193)	(0.193)
Political Efficacy	0.060	0.056	0.057	0.058	0.057	0.055	0.055	0.056	0.054
	(0.044)	(0.044)	(0.044)	(0.044)	(0.044)	(0.044)	(0.044)	(0.044)	(0.044)
Political Interest	0.531***	0.536***	0.531***	0.529***	0.531***	0.534***	0.529***	0.533***	0.534***
	(0.052)	(0.052)	(0.052)	(0.052)	(0.052)	(0.052)	(0.052)	(0.052)	(0.052)
Worship Attendance	0.076***	0.076***	0.077***	0.077***	0.077***	0.077***	0.078***	0.075***	0.078***
	(0.026)	(0.026)	(0.026)	(0.026)	(0.026)	(0.026)	(0.026)	(0.026)	(0.026)
Party ID	−0.117	−0.107	−0.119	−0.120	−0.117	−0.117	−0.117	−0.125	−0.119
	(0.091)	(0.091)	(0.091)	(0.091)	(0.091)	(0.091)	(0.090)	(0.091)	(0.091)
Female	−0.045	−0.046	−0.051	−0.046	−0.049	−0.050	−0.044	−0.048	−0.052
	(0.093)	(0.093)	(0.093)	(0.093)	(0.093)	(0.093)	(0.093)	(0.093)	(0.093)
Age 18-29	−0.051	−0.044	−0.057	−0.053	−0.050	−0.055	−0.054	−0.056	−0.056
	(0.173)	(0.173)	(0.173)	(0.173)	(0.174)	(0.174)	(0.173)	(0.173)	(0.174)
Age 30-39	−0.119	−0.103	−0.120	−0.118	−0.121	−0.119	−0.105	−0.118	−0.119
	(0.165)	(0.166)	(0.166)	(0.166)	(0.166)	(0.166)	(0.165)	(0.165)	(0.166)
Age 40-64	0.052	0.066	0.056	0.052	0.059	0.057	0.060	0.058	0.058
	(0.158)	(0.158)	(0.158)	(0.158)	(0.158)	(0.158)	(0.157)	(0.158)	(0.158)
Education	−0.017	−0.012	−0.019	−0.018	−0.016	−0.017	−0.013	−0.016	−0.018
	(0.046)	(0.046)	(0.046)	(0.046)	(0.046)	(0.046)	(0.046)	(0.046)	(0.046)
Income < 40K	−0.142	−0.135	−0.144	−0.148	−0.143	−0.140	−0.141	−0.138	−0.140
	(0.128)	(0.128)	(0.128)	(0.128)	(0.128)	(0.128)	(0.128)	(0.128)	(0.128)
Income 40-70K	0.015	0.010	0.016	0.013	0.015	0.016	0.005	0.018	0.019
	(0.107)	(0.107)	(0.107)	(0.107)	(0.107)	(0.107)	(0.107)	(0.107)	(0.107)
Contact X Disc	−0.532**	−0.430**	−0.362*	−0.427**	−0.367*	−0.318	−0.397***	−0.444**	−0.258
	(0.215)	(0.172)	(0.198)	(0.202)	(0.201)	(0.216)	(0.145)	(0.200)	(0.197)
Constant	−0.180	−0.220	−0.159	−0.163	−0.171	−0.162	−0.203	−0.170	−0.158
	(0.264)	(0.265)	(0.264)	(0.264)	(0.264)	(0.264)	(0.264)	(0.264)	(0.264)
Observations	1,158	1,158	1,158	1,158	1,158	1,158	1,158	1,158	1,158
R^2	0.216	0.216	0.214	0.215	0.214	0.213	0.217	0.215	0.213
Adjusted R^2	0.201	0.201	0.199	0.199	0.199	0.198	0.202	0.200	0.198

Note: *p<0.1; **p<0.05; ***p<0.01

D Voting among racial subgroups

Figure A4: **The impact of a politicized group identity and institutional contact on self-reported voting by race**

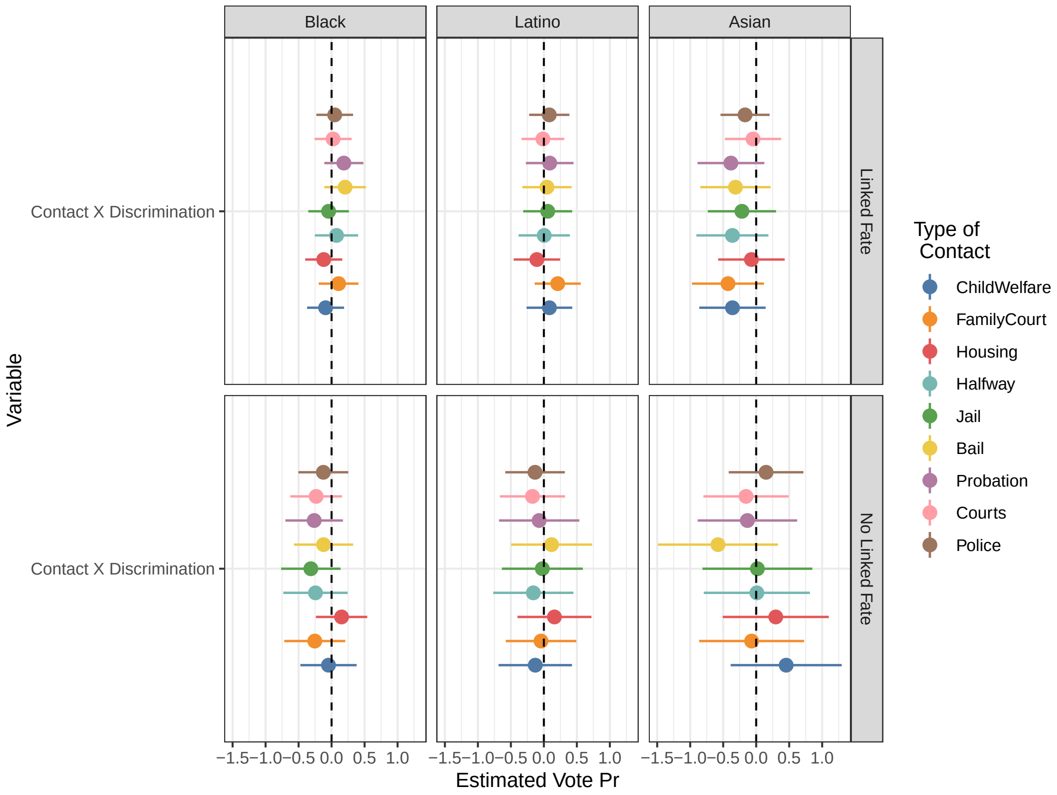

E Figures and tables associated with additional Robustness Checks

Figure A5: The impact of a politicized identity and institutional contact on items in the participation battery

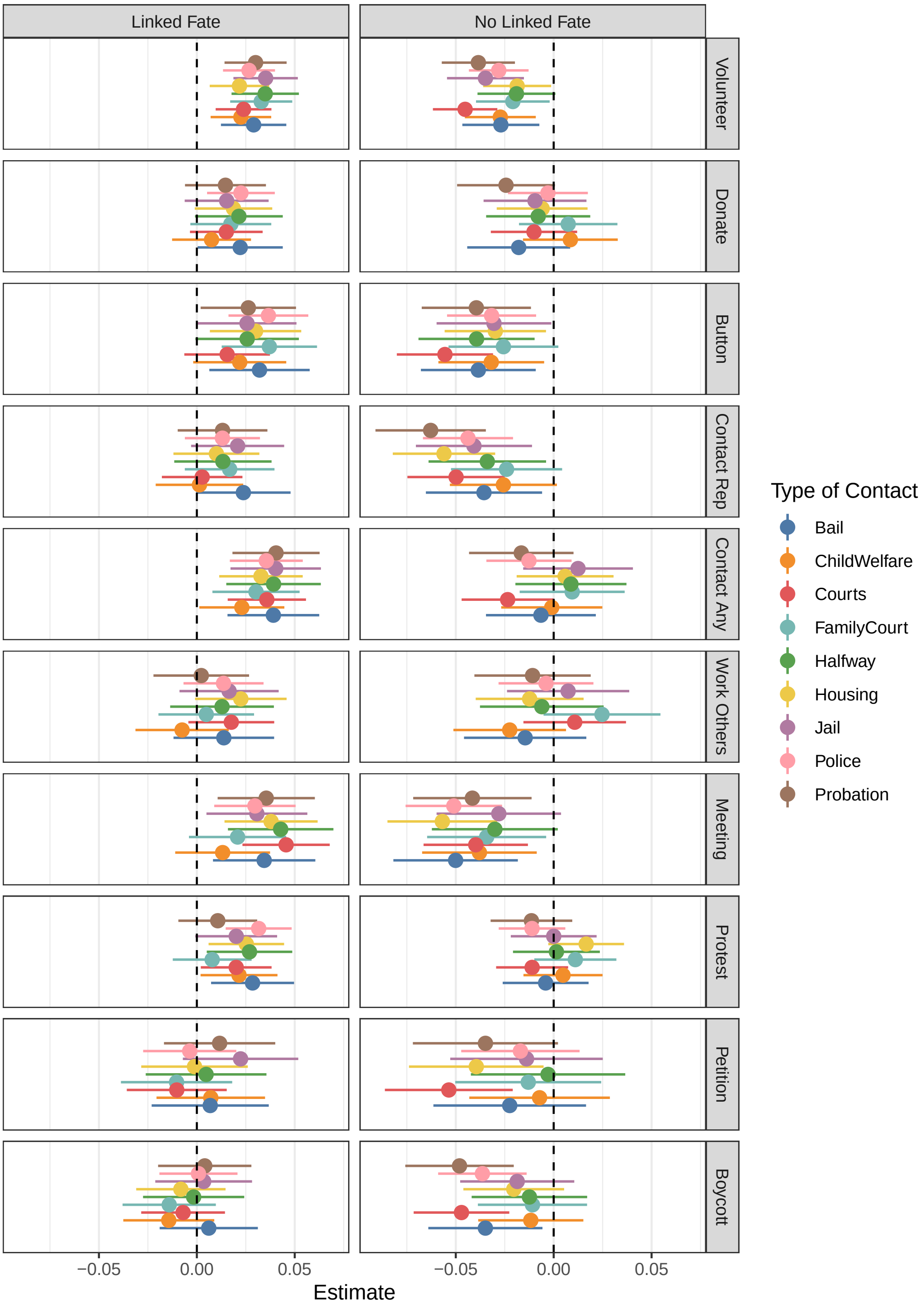

Table A11: Moderation analysis: The impact of discrimination and an index of institutional contacts on participation, among those with and without linked fate

	Dependent variable:	
	Political Participaton	
	Linked Fate	No Linked Fate
	(1)	(2)
Discrimination	0.531***	0.511***
	(0.067)	(0.066)
Contact Index	0.069***	0.070***
	(0.008)	(0.007)
Political Efficacy	0.108***	0.058**
	(0.026)	(0.026)
Political Interest	0.895***	0.538***
	(0.035)	(0.032)
Worship Attendance	0.048***	0.071***
	(0.016)	(0.016)
Party ID	0.089	−0.078
	(0.062)	(0.060)
Female	0.020	−0.085
	(0.061)	(0.061)
Age 40-64	−0.171	−0.117
	(0.122)	(0.113)
Age 30-39	−0.337***	−0.357***
	(0.123)	(0.112)
Age 18-29	−0.479***	−0.123
	(0.117)	(0.105)
Education	0.226***	0.096***
	(0.030)	(0.029)
Income < 40K	−0.534***	−0.256***
	(0.080)	(0.082)
Income 40-70K	−0.153**	0.060
	(0.076)	(0.077)
Asian	−0.379***	−0.150*
	(0.075)	(0.078)
Latino	0.152**	−0.040
	(0.071)	(0.071)
Contact X Disc	0.028***	−0.035***
	(0.010)	(0.012)
Constant	−0.568***	−0.086
	(0.193)	(0.183)
Observations	5,656	3,442
R^2	0.241	0.184
Adjusted R^2	0.239	0.180
Note:	*p<0.1; **p<0.05; ***p<0.01	

Table A12: Moderation analysis: The impact of discrimination and institutional contact on participation, among Asians and Latinos with a strong panethnic identity

	Dependent variable:								
	Political Participation								
	Bail	Court	Family	Halfway	Housing	Jail	Police	Probation	Child Welfare
	(1)	(2)	(3)	(4)	(5)	(6)	(7)	(8)	(9)
Contact	−0.100	−0.129	−0.255**	−0.080	0.014	−0.118	0.267***	−0.223	−0.002
	(0.154)	(0.113)	(0.126)	(0.144)	(0.122)	(0.157)	(0.088)	(0.152)	(0.129)
Discrimination	0.606***	0.583***	0.642***	0.614***	0.609***	0.591***	0.532***	0.608***	0.644***
	(0.083)	(0.087)	(0.083)	(0.082)	(0.083)	(0.082)	(0.090)	(0.083)	(0.083)
Child Welfare	0.047	0.040	0.046	0.050	0.048	0.040	0.039	0.043	
	(0.103)	(0.103)	(0.103)	(0.103)	(0.103)	(0.103)	(0.103)	(0.103)	
Family Court	−0.204*	−0.198*		−0.203*	−0.211**	−0.209**	−0.194*	−0.210**	−0.204*
	(0.106)	(0.106)		(0.106)	(0.106)	(0.106)	(0.106)	(0.106)	(0.106)
Housing	0.146	0.142	0.140	0.144		0.153	0.162	0.142	0.143
	(0.100)	(0.100)	(0.100)	(0.100)		(0.100)	(0.100)	(0.100)	(0.100)
Halfway	0.060	0.068	0.068		0.072	0.080	0.075	0.068	0.068
	(0.123)	(0.123)	(0.123)		(0.123)	(0.123)	(0.123)	(0.123)	(0.123)
Jail	0.137	0.136	0.138	0.139	0.141		0.112	0.140	0.139
	(0.126)	(0.126)	(0.126)	(0.126)	(0.126)		(0.126)	(0.126)	(0.126)
Bail		0.072	0.063	0.052	0.063	0.068	0.068	0.059	0.062
		(0.133)	(0.133)	(0.132)	(0.132)	(0.132)	(0.132)	(0.132)	(0.133)
Probation	−0.074	−0.065	−0.067	−0.072	−0.077	−0.069	−0.068		−0.065
	(0.129)	(0.129)	(0.130)	(0.129)	(0.130)	(0.129)	(0.129)		(0.130)
Courts	0.023		0.018	0.018	0.015	0.024	0.016	0.021	0.018
	(0.087)		(0.087)	(0.087)	(0.087)	(0.087)	(0.087)	(0.087)	(0.087)
Police	0.442***	0.442***	0.441***	0.444***	0.447***	0.436***		0.442***	0.440***
	(0.065)	(0.065)	(0.065)	(0.065)	(0.065)	(0.065)		(0.065)	(0.065)
Political Efficacy	0.067*	0.068*	0.068*	0.067*	0.067*	0.065*	0.067*	0.067*	0.067*
	(0.036)	(0.036)	(0.036)	(0.036)	(0.036)	(0.036)	(0.036)	(0.036)	(0.036)
Political Interest	0.800***	0.798***	0.799***	0.799***	0.800***	0.801***	0.800***	0.800***	0.799***
	(0.048)	(0.048)	(0.048)	(0.048)	(0.048)	(0.048)	(0.048)	(0.048)	(0.048)
Worship Attendance	0.005	0.005	0.006	0.006	0.006	0.005	0.007	0.006	0.006
	(0.022)	(0.022)	(0.022)	(0.022)	(0.022)	(0.022)	(0.022)	(0.022)	(0.022)
Party ID	0.034	0.027	0.034	0.034	0.032	0.036	0.028	0.034	0.034
	(0.084)	(0.084)	(0.084)	(0.084)	(0.084)	(0.084)	(0.084)	(0.084)	(0.084)
Female	−0.013	−0.011	−0.009	−0.012	−0.011	−0.009	−0.015	−0.012	−0.009
	(0.088)	(0.088)	(0.088)	(0.088)	(0.088)	(0.088)	(0.088)	(0.088)	(0.088)
Age 40-64	0.199	0.195	0.190	0.192	0.195	0.198	0.200	0.195	0.191
	(0.181)	(0.181)	(0.181)	(0.181)	(0.181)	(0.181)	(0.181)	(0.181)	(0.181)
Age 30-39	−0.033	−0.036	−0.030	−0.034	−0.031	−0.038	−0.035	−0.034	−0.030
	(0.181)	(0.181)	(0.181)	(0.181)	(0.181)	(0.181)	(0.181)	(0.181)	(0.181)
Age 18-29	−0.011	−0.014	−0.012	−0.012	−0.010	−0.008	−0.007	−0.012	−0.011
	(0.176)	(0.176)	(0.176)	(0.176)	(0.176)	(0.176)	(0.176)	(0.176)	(0.176)
Education	0.218***	0.215***	0.214***	0.216***	0.215***	0.218***	0.213***	0.217***	0.214***
	(0.040)	(0.040)	(0.040)	(0.040)	(0.040)	(0.040)	(0.039)	(0.040)	(0.040)
Income < 40K	−0.525***	−0.528***	−0.520***	−0.522***	−0.525***	−0.531***	−0.530***	−0.525***	−0.521***
	(0.111)	(0.111)	(0.111)	(0.111)	(0.111)	(0.111)	(0.111)	(0.111)	(0.111)
Income 40-70K	−0.103	−0.109	−0.102	−0.104	−0.101	−0.106	−0.101	−0.101	−0.102
	(0.104)	(0.104)	(0.104)	(0.104)	(0.104)	(0.104)	(0.104)	(0.104)	(0.104)
Latino	0.411***	0.409***	0.410***	0.411***	0.410***	0.417***	0.403***	0.412***	0.410***
	(0.086)	(0.086)	(0.086)	(0.086)	(0.086)	(0.086)	(0.086)	(0.086)	(0.086)
Contact X Disc	0.281**	0.229**	0.085	0.258*	0.223*	0.387***	0.286***	0.263**	0.073
	(0.137)	(0.113)	(0.125)	(0.136)	(0.123)	(0.140)	(0.097)	(0.131)	(0.124)
Constant	−1.077***	−1.046***	−1.091***	−1.074***	−1.072***	−1.068***	−1.021***	−1.076***	−1.092***
	(0.253)	(0.254)	(0.253)	(0.253)	(0.254)	(0.253)	(0.254)	(0.253)	(0.253)
Observations	2,741	2,741	2,741	2,741	2,741	2,741	2,741	2,741	2,741
R^2	0.234	0.234	0.233	0.234	0.234	0.235	0.235	0.234	0.233
Adjusted R^2	0.228	0.228	0.227	0.228	0.227	0.229	0.229	0.228	0.227

Note: *p<0.1; **p<0.05; ***p<0.01

Table A13: Moderation analysis: The impact of discrimination and institutional contact on participation, among Asians and Latinos without a strong panethnic identity

	Dependent variable:								
	Political Participation								
	Bail	Court	Family	Halfway	Housing	Jail	Police	Probation	Child Welfare
	(1)	(2)	(3)	(4)	(5)	(6)	(7)	(8)	(9)
Contact	−0.079	0.705***	−0.482	1.090***	−0.022	−0.026	0.605**	−0.752*	−0.156
	(0.287)	(0.263)	(0.326)	(0.361)	(0.359)	(0.317)	(0.239)	(0.417)	(0.265)
Discrimination	0.484*	0.724***	0.372	0.402	0.384	0.352	0.708**	0.365	0.303
	(0.251)	(0.267)	(0.253)	(0.248)	(0.249)	(0.256)	(0.276)	(0.255)	(0.253)
Child Welfare	−0.094	−0.195	−0.076	−0.074	−0.074	−0.077	−0.106	−0.074	
	(0.248)	(0.250)	(0.248)	(0.248)	(0.251)	(0.248)	(0.247)	(0.248)	
Family Court	−0.382	−0.392		−0.451	−0.462	−0.473	−0.385	−0.465	−0.503*
	(0.302)	(0.291)		(0.298)	(0.297)	(0.296)	(0.293)	(0.294)	(0.297)
Housing	−0.050	−0.101	−0.012	−0.030		−0.020	−0.113	−0.010	0.056
	(0.347)	(0.344)	(0.348)	(0.353)		(0.346)	(0.346)	(0.348)	(0.356)
Halfway	1.098***	1.203***	1.059***		1.065***	1.062***	1.187***	1.051***	1.020***
	(0.330)	(0.330)	(0.331)		(0.329)	(0.329)	(0.331)	(0.335)	(0.333)
Jail	−0.043	−0.101	0.004	−0.006	0.001		−0.108	0.007	−0.005
	(0.310)	(0.306)	(0.308)	(0.311)	(0.308)		(0.309)	(0.308)	(0.307)
Bail		−0.203	−0.134	−0.142	−0.133	−0.121	−0.145	−0.124	−0.112
		(0.280)	(0.282)	(0.285)	(0.284)	(0.285)	(0.280)	(0.286)	(0.283)
Probation	−0.701*	−0.694*	−0.716*	−0.704*	−0.718*	−0.728*	−0.831**		−0.758*
	(0.385)	(0.381)	(0.385)	(0.393)	(0.387)	(0.387)	(0.386)		(0.388)
Courts	0.382*		0.362	0.362	0.360	0.362	0.471**	0.356	0.380*
	(0.229)		(0.228)	(0.228)	(0.228)	(0.228)	(0.232)	(0.229)	(0.229)
Police	0.366*	0.413*	0.377*	0.369*	0.376*	0.387*		0.384*	0.397*
	(0.215)	(0.214)	(0.217)	(0.217)	(0.218)	(0.219)		(0.220)	(0.217)
Political Efficacy	−0.004	−0.001	−0.011	−0.010	−0.011	−0.012	−0.004	−0.011	−0.016
	(0.077)	(0.076)	(0.077)	(0.077)	(0.077)	(0.077)	(0.076)	(0.077)	(0.077)
Political Interest	0.459***	0.432***	0.468***	0.466***	0.467***	0.469***	0.430***	0.468***	0.474***
	(0.096)	(0.096)	(0.097)	(0.096)	(0.096)	(0.096)	(0.097)	(0.096)	(0.096)
Worship Attendance	0.237***	0.233***	0.236***	0.237***	0.237***	0.236***	0.234***	0.237***	0.234***
	(0.063)	(0.063)	(0.063)	(0.063)	(0.063)	(0.063)	(0.063)	(0.063)	(0.063)
Party ID	−0.091	−0.060	−0.105	−0.098	−0.101	−0.105	−0.090	−0.101	−0.110
	(0.208)	(0.206)	(0.210)	(0.209)	(0.209)	(0.209)	(0.206)	(0.208)	(0.208)
Female	−0.102	−0.103	−0.102	−0.105	−0.103	−0.102	−0.128	−0.102	−0.099
	(0.203)	(0.201)	(0.204)	(0.204)	(0.204)	(0.204)	(0.202)	(0.204)	(0.203)
Age 40-64	0.053	0.026	0.037	0.043	0.040	0.034	0.060	0.042	0.025
	(0.397)	(0.393)	(0.398)	(0.398)	(0.398)	(0.398)	(0.394)	(0.398)	(0.397)
Age 30-39	0.122	0.121	0.127	0.124	0.125	0.133	0.122	0.126	0.130
	(0.394)	(0.390)	(0.395)	(0.395)	(0.396)	(0.396)	(0.392)	(0.395)	(0.395)
Age 18-29	0.541	0.542	0.555	0.553	0.555	0.558	0.547	0.557	0.557
	(0.371)	(0.368)	(0.372)	(0.372)	(0.372)	(0.372)	(0.369)	(0.372)	(0.371)
Education	−0.114	−0.108	−0.114	−0.115	−0.115	−0.114	−0.116	−0.115	−0.111
	(0.088)	(0.088)	(0.089)	(0.089)	(0.089)	(0.089)	(0.088)	(0.089)	(0.089)
Income < 40K	−0.009	−0.006	0.012	0.008	0.010	0.013	−0.058	0.008	0.018
	(0.267)	(0.264)	(0.268)	(0.268)	(0.267)	(0.267)	(0.267)	(0.267)	(0.267)
Income 40-70K	−0.322	−0.340	−0.301	−0.307	−0.304	−0.299	−0.356	−0.303	−0.301
	(0.264)	(0.261)	(0.265)	(0.264)	(0.264)	(0.264)	(0.263)	(0.264)	(0.263)
Latino	−0.094	−0.067	−0.089	−0.090	−0.091	−0.090	−0.039	−0.092	−0.087
	(0.202)	(0.200)	(0.203)	(0.203)	(0.203)	(0.203)	(0.202)	(0.203)	(0.202)
Contact X Disc	−0.391	−0.819**	0.050	−0.057	0.017	0.121	−0.710**	0.081	0.302
	(0.374)	(0.324)	(0.319)	(0.333)	(0.368)	(0.347)	(0.331)	(0.363)	(0.349)
Constant	0.218	0.173	0.239	0.237	0.240	0.242	0.233	0.243	0.251
	(0.555)	(0.549)	(0.555)	(0.556)	(0.556)	(0.555)	(0.551)	(0.556)	(0.555)
Observations	287	287	287	287	287	287	287	287	287
R^2	0.279	0.293	0.276	0.276	0.276	0.277	0.289	0.276	0.278
Adjusted R^2	0.216	0.232	0.213	0.213	0.213	0.213	0.226	0.213	0.215

Note: *p<0.1; **p<0.05; ***p<0.01
